# Butterfly oviposition preference is not related to larval performance on a polyploid herb

**DOI:** 10.1002/ece3.2067

**Published:** 2016-03-20

**Authors:** Malin A. E. König, Christer Wiklund, Johan Ehrlén

**Affiliations:** ^1^ Department of EcologyEnvironment and Plant SciencesStockholm UniversitySE106 91StockholmSweden; ^2^ Department of ZoologyStockholm UniversitySE106 91StockholmSweden

**Keywords:** *Anthocharis cardamines*, *Cardamine pratensis*, cytotype, herbivory, host plant quality, naive adaptationist hypothesis

## Abstract

The preference–performance hypothesis predicts that female insects maximize their fitness by utilizing host plants which are associated with high larval performance. Still, studies with several insect species have failed to find a positive correlation between oviposition preference and larval performance. In the present study, we experimentally investigated the relationship between oviposition preferences and larval performance in the butterfly *Anthocharis cardamines*. Preferences were assessed using both cage experiments and field data on the proportion of host plant individuals utilized in natural populations. Larval performance was experimentally investigated using larvae descending from 419 oviposition events by 21 females on plants from 51 populations of two ploidy types of the perennial herb *Cardamine pratensis*. Neither ploidy type nor population identity influenced egg survival or larval development, but increased plant inflorescence size resulted in a larger final larval size. There was no correlation between female oviposition preference and egg survival or larval development under controlled conditions. Moreover, variation in larval performance among populations under controlled conditions was not correlated with the proportion of host plants utilized in the field. Lastly, first instar larvae added to plants rejected for oviposition by butterfly females during the preference experiment performed equally well as larvae growing on plants chosen for oviposition. The lack of a correlation between larval performance and oviposition preference for *A. cardamines* under both experimental and natural settings suggests that female host choice does not maximize the fitness of the individual offspring.

## Introduction

The quality of host plants as food for insect larvae, e.g., in terms of nutritional quality and presence of plant defenses, can vary greatly both between plant species and among individuals of the same species (e.g., Awmack and Leather 2002). Host plant individuals also differ with regard to many other factors that might affect larval development and survival, e.g., intensity of competition, predation, parasitism, light availability, and temperature (Scriber and Slansky 1981). Since first instar insect larvae are relatively immobile and often unable to switch from one host plant to another, the female's choice of plant for oviposition is crucial for the fitness of the individual larva. The larvae from females which are able to correctly assess plant quality via plant traits should gain an advantage over females which lack this ability. This suggests that there is strong selection for female ability to evaluate the quality of the host plant. The preference–performance hypothesis, also known as the “mother knows best hypothesis” or the “naive adaptationist hypothesis”, predicts that females will maximize their own fitness by utilizing host plants which maximize larval performance (Jaenike 1978; Gripenberg et al. 2010). Although the majority of studies investigating oviposition choice and larval performance indeed report positive relationships between oviposition preference and larval performance (Craig et al. 1989; Nylin and Janz 1993; Gripenberg et al. 2010), accumulating evidence indicates that not all insect species choose the best host plants for their offspring (Scheirs et al. 2000; Scheirs 2002; Refsnider and Janzen 2010). Several explanations for such a lack of positive correlation between oviposition preference and larval performance have been suggested; e.g., rarity of optimal plant hosts, selection for enemy free space rather than plant quality and unpredictability among years in survival rate on specific host plants (Thompson 1988; Wiklund and Friberg 2009). Another explanation for lack of positive correlations is that females stay close to plants or habitats which maximize their own survival and fecundity rather than the fitness of the individual offspring (Mayhew 2001; Scheirs et al. 2000; Garcia‐Robledo and Horvitz 2012). Time‐limitation should also decrease the correlation between female oviposition choice and larval performance as short‐lived females with many eggs cannot spend too much time on finding the optimal host (Rosenheim et al. 2008). The benefit of a higher number of offspring might then counterbalance the costs of reduced development rate and survival of the individual larvae (Courtney 1981; Scheirs et al. 2000; Mayhew 2001). Generalists are often considered less able than specialists at predicting individual host plant quality, since the ability to recognize numerous host species is expected to be traded off against the ability to evaluate the nutritional quality of the specific plant individual (Wiklund 1981; Janz 2003; Liu et al. 2012). Although theories about relationships between host preferences, plant quality, and larval performance apply equally to among and within host plant species choices, studies have often focused on between host species choices while studies of choices within species are less common. Still, we know that in many systems insect oviposition rates vary within species among populations (Wiklund 1974a; Thompson 1988; Singer and McBride 2012). One possible cause of such among‐population variation in oviposition rates, is that plant populations may differ in ploidy levels and thus will, despite having similar physiological traits, differ in number of copies of all genes, including genes linked to resistance against herbivory. Studies have shown that oviposition rates differ between different ploidy types (Thompson et al. 1997; Nuismer and Thompson 2001; Münzbergová 2006; Münzbergová et al. 2015), but none have, to our knowledge, investigated if larval performance was affected by the ploidy level of the host plant.

To test whether the correlation between oviposition preference and larval performance differ among host plant populations and ploidy types, we used a model system consisting of the butterfly *Anthocharis cardamines* (L.) and 51 populations of two ploidy types of the perennial herb *Cardamine pratensis* (L.). *Anthocharis cardamines* is a generalist butterfly which utilizes a broad range of Brassicaceae species (Courtney 1981). The most preferred host species in Sweden is *C*. *pratensis* (Navarro‐Cano et al. 2015), an autopolyploid which occurs as both tetraploid and octoploid in Sweden (Arvanitis et al. 2007). In the field, female *A. cardamines* prefer tetraploid populations over octoploid populations, but in sympatric populations octoploid individuals are preferred over tetraploid (Arvanitis et al. 2008). Previous studies have also demonstrated that oviposition rates differ among populations of the same ploidy type, suggesting that differences in oviposition preference are linked to habitat differences rather than innate plant traits (Arvanitis et al. 2007). In this study, our aim was to examine if observed preference patterns in the field and under controlled environmental conditions are associated with differences in larval performance. We asked the following specific questions: 1. Does egg survival and larval development differ between ploidy types, and among populations within ploidy types? 2. What plant traits influence larval performance? 3. Is larval performance positively correlated with female host plant choice under controlled environmental conditions? and 4. Is larval performance positively correlated with female host plant choice in the field?

## Materials and Methods

### Study system

The orange tip butterfly, *A. cardamines* (Pieridae), is obligately univoltine and flies in a single generation during May – June. Females are good fliers and often pass through forest corridors in their search for suitable host plants, but oviposit upon plants growing in open areas. Since potential host plants are localized by visual cues, *A. cardamines* only oviposit upon flowering plants (Dempster 1997). The female oviposits a single egg in the inflorescence together with an oviposition deterrent pheromone (Dempster 1992), and within 7–10 days the egg hatches and the larva begins to feed on buds, flowers, and siliques. Previous studies have shown that female *A. cardamines* do not always choose the plant species on which the larvae develop best for oviposition (Courtney 1981), but little is known about the relationships between female preference and larval performance within host species.

*Cardamine pratensis* (Brassicaceae) is a perennial herb, which occurs in different ploidy levels throughout Europe (Lövkvist 1956). In southern Sweden, both tetraploids and octoploids are commonly found. *Anthocharis cardamines* is one of the main herbivores on *C. pratensis*, and a successful oviposition and larval development results in a complete seed loss for the plant (Arvanitis et al. 2007). The two ploidy types are possible to separate in the field and assessments of ploidy level for plants from the study populations based on morphological traits have been confirmed through flow cytometry studies (Arvanitis et al. 2008). Hosting a larva also leads to fitness losses the following flowering season; both tetraploids and octoploids produce fewer flowers and are less likely to flower the year after butterfly attack (König et al. 2014a).

In a previous experiment with this system, the effects of plant traits in the two cytotypes of *C. pratensis* versus environmental context on oviposition preferences by *A. cardamines* were examined (König et al. 2014b). The results showed that octoploids were preferred over tetraploids under controlled conditions while tetraploids are more frequently attacked in the field. This suggests that octoploids provide better resources but that tetraploids grow in environments that are better for larvae or adults.

### Study design

Plant material for preference–performance experiments was collected in the summer of 2009 from 1 to 4 plants in 24 tetraploid and 27 octoploid populations of *C. pratensis*, located within a 95 km^2^ area in the parish of Ludgo, Sweden. The *C. pratensis* populations were geographically separated but still close enough to allow female *A. cardamines* to fly between them during their search for host plants. This would in theory allow a female to choose where to oviposit her eggs in the populations within the area, exerting stronger selection in preferred populations. The collected leaflets were potted in sowing soil (S‐jord from Hasselfors Garden) to create cloned ramets of each individual plant. After 2–3 weeks, the small plants were planted in larger pots and grown in the greenhouse for another month. The plants were then kept in the common garden from August 2009 – May 2010.

The butterflies used in the experiment were collected as eggs or young larvae on wild octoploid *C. pratensis* from populations located in the vicinity of Stockholm in 2008. These plant populations were located some 100 km from the *C. pratensis* populations used for the preference/performance analysis. The larvae were reared in the laboratory at the Department of Zoology, Stockholm University, on octoploid *C. pratensis* from the plant populations on which the eggs/larvae were collected. After pupation, the pupae were kept at 23°C until mid‐September and then overwintered at 0°C until early May. Females were marked with a permanent marker upon eclosion and mated in cages in the laboratory. In total, 22 females eclosed in 2009. After mating, the females were kept in a cold room maintained at 10°C, until they were used in the experiment. The females were thus naive to Brassicaceae plants, in terms of adult experience, at the start of the experimental trials. In butterflies, it is generally the case that the host plant of the larva does not influence the host plant preference of the adult females (Wiklund 1974b; Janz et al. 2009; but see Cahenzli et al. 2015 for recent evidence that the nutritional quality of the host plant may have carryover effects from larva to adult).

In 2010, 448 of the cloned ramets flowered and were first used for an oviposition preference experiment (König et al. 2014b), and afterwards 419 of these 448 plants were used for a larval performance experiment. Because the oviposition preference experiment aimed to remove the effects of habitat differences among populations and individuals as well as the effects of flowering phenology, flowering plants of similar phenological state were used. Up to four flowering ramets of each genet were haphazardly chosen and divided into groups consisting of eight tetraploids and eight octoploids. Each group was placed in a 2.5 × 2.5 × 1.8 m cage with one mated *A. cardamines* female. Before the experiment, the number of flowers, flower diameter, date of first open flower, and height and basal diameter of the inflorescence shoot were recorded for each plant. The time that elapsed from the release of the female to oviposition on a given plant was used as a measurement of butterfly preference for the plant individual. This procedure allowed us to establish a preference hierarchy and the plant selected before another plant would always receive a higher preference value, irrespective of the outcome of subsequent trials. Hence, although the proportion of the less preferred ploidy type increased during a trial, this did not compromise our ambition to establish a hierarchy of butterfly preference for one ploidy type over the other. The experiment was terminated when all plants had been oviposited upon or when the sun began to descend. If less than six individuals had received an egg during the first day, the experimental trial was continued the next day. Each oviposition time was standardized by subtracting the mean oviposition time of the experimental trial and dividing the difference by the standard deviation of the trial, leaving each trial with a mean of zero and a standard deviation of one. Plants that did not receive an egg were assigned the time when the experiment was ended and were included in the standardization of the trials. The standardization within experimental trials means that mean differences in time to oviposition among cage trials do not influence our estimate of oviposition preference. In total, 37 experimental replicates involving 51 plant populations, 164 genets and 448 cloned ramets were carried out (for more details of the experimental setup see König et al. 2014b).

To examine the larval performance in this study, the plants with butterfly eggs from the preference experiment were placed in a common garden in water filled trays under a thin fabric. The trays acted both to keep the soil moist and as a moat, hindering *A. cardamines* larvae from moving between plants. The fabric protected both the plants from oviposition by wild butterfly females in the area and larvae from predation. The plants were monitored once a week, and each time the length of the larvae was recorded and used as an estimate of larval performance. Monitoring larval survival and assessing larval performance in nature, or in a natural setting as in our experiment, is notoriously difficult for the obvious reason that larvae should be disturbed as little as possible and because the fully grown larvae leave their host plant to search for a pupation site once they have completed larval development. The only way to assess larval performance in a natural setting without handling them is to measure the rate at which the larvae grow, which can be assessed by measuring the length of larvae (cf Frohawk (1914) documenting how *A. cardamines* larvae increase in length over time during their development). The logic behind using final larval length as a measure of performance is that larvae might disappear from a host plant for three reasons: (1) to search for a pupation site, (2) to find a new host plant to continue larval feeding, and (3) because larvae have died, and we expect the probabilities of each of these three events to depend on larval size. For all of these three cases, performance/fitness is likely to increase with larval size. (1) Because larvae leave the host plant to search for a pupation site when they have completed development are the largest, the larger the “final size” of the larvae that we measured the closer they are to the time when they leave the host plant to pupate. (2) Because larval mobility increases with size (first instar larvae are incapable of leaving the plant on which they hatched, they are simply too small, whereas larvae, especially from the 3rd instar onwards are increasingly mobile) the likelihood of finding a new host plant increases with larval size. (3) For the same reason, (larval mobility increasing with size), we expect the proportion of larvae that have died decreases with larval size. Hence, the critical assumption of our study is that a larger size means a higher fitness. For this to be true, it is sufficient that the fraction of larvae that leave the plant that will eventually pupate will increase with size, irrespective of whether this occurs because the larvae pupates directly after leaving the plant, or if pupation takes place only after feeding on an additional host plant.

Twenty‐six plants were completely rejected for oviposition by the females in the preference experiment. To investigate if larvae performed worse on the rejected plants than those accepted by the females, young first instar larvae hatched from eggs oviposited upon octoploid *C. pratensis* in the laboratory were added to rejected plants.

To examine if larval performance was positively correlated with female host plant selection in the field, i.e., if females prefer populations with host plant individuals that are optimal for larval survival and growth, we estimated female preference in the field as the mean proportion of plants oviposited upon in a population. Host plant use was estimated in a subset of the 51 study populations, ten tetraploid and eleven octoploid, during 2009, 2010, 2011, 2012 and 2013. Every year, up to 30 flowering plants were marked and examined once a week during the flowering period for the presence or absence of *A. cardamines* eggs. The among‐year mean of proportion of plants oviposited upon was used as a measure of host plant use.

### Statistical analyses

All the statistical analyses were performed in R 3.2.3 (R Core Team 2015). Data from a previous study (König et al. 2014b, Appendix S1 table S1‐1) on the plant traits flower size and inflorescence size, and oviposition preference rankings were used in analyses to assess the relationships with larval performance examined in this study. The inflorescence shoot volume was estimated as the volume of a cylinder using the diameter and height of the inflorescence shoot. The inflorescence shoot volume was log_10‐_transformed and number of flowers square root‐transformed to achieve normal distributions. Inflorescence shoot volume (log_10_‐transformed) and number of flowers (square root‐transformed) were highly positively correlated for both ploidy types. Thus, the first principal component of the two variables was extracted from a correlation matrix using the prcomp‐function in R 2.15.3. This first principal component explained 78% of the variance in tetraploids and 74% of the variance in octoploids. It was thus used for analyses and will be referred to as plant inflorescence size in the following.

The data set contained two random factors, butterfly female and the nested structure of multiple plants within genets, multiple genets within population and multiple populations within ploidy type. Models including multiple random factors yielded results that were very similar to those of generalized linear models (GLMs) without random effects. The results of both the GLMs and the corresponding nested models including random effects, are provided in the supplementary material (Supplementary material Appendix S2). For clarity, we refer only the results of the GLMs in the text.

To examine if the probability of egg survival, i.e., if an egg hatched or not, differed between the two ploidy types, we used a GLM with family set to binomial including only “ploidy type”. In this analysis, a genet had to be represented by at least two plants, a population by at least two genets and butterfly females by at least two eggs to be included to allow variance within the random effect‐groups, leaving 419 plants of the 448 plants used in the preference experiment, representing 163 genets, 51 populations and eggs from 21 females.

To investigate if the length of the larvae before disappearance (hereon referred to as final size) differed between the two ploidy types, we used GLMs including only “ploidy type”. To examine also the effect of the measured plant traits, we used GLMs including the plant traits “flower diameter”, “plant inflorescence size” and “phenology”, in addition to “ploidy type”. In both of these two analyses, a genet had to be represented by at least two plants, a population by at least two genets and females by at least two hatched larvae to be included, leaving 173 plants, representing 75 genets, 30 populations and eggs from 18 females. Although plants which had flowered for approximately the same number of days were chosen for the cage preference experiment, some variation in phenology remained which may have affected larval development. Phenological state was therefore included as a covariate in the initial model. However, phenological state did not have a significant effect on larval size.

To investigate if female preference under controlled conditions was related to egg survival, a GLM with family set to binomial was used. “Egg survival” was set as response variable, “female oviposition preference”, “ploidy type” and the interaction between the two variables were used as explanatory variables.

To investigate if female preference under controlled conditions was related to larval performance, final larval size was correlated to the preference values achieved in the cage preference experiment. “Larval final size” was set as response variable, “female preference”, “ploidy type” and the interaction term were used as explanatory variables.

To test the hypothesis that the 26 plants rejected for oviposition in the preference experiment were avoided by females for reasons coupled to larval development, a first instar larva hatched in the laboratory was added to rejected plants. To test if the final size of added larvae was smaller than the final size of larvae developing on plants that were chosen by females, we used a GLM with “final larval size” as the response variable, and “larval treatment”, i.e., oviposited or added larvae, “ploidy type” and the interaction term as explanatory variables (for data see Apendix S1 table S1‐2). In this analysis 307 plants, representing 159 genets, and 51 populations were included.

To investigate if larval performance in the experiment was related to host plant use in the field, we used a linear model with “population mean larval size” as the response variable, and “mean host plant use across years”, “ploidy type” and the interaction term as explanatory variables (for data see Apendix S1 table S1‐3).

## Results

Egg survival ranged from 20 to 100% across the 51 populations and mean larval final size ranged from 0.30 to 2.75 cm. There was no difference between tetraploid and octoploid individuals in the proportion of eggs hatching (*P = *0.34, *z*‐value = 0.96, Table S2‐1) or in final larval size (*P *=* *0.27, *t*‐value = −1.11, Table S2‐2).

**Figure 1:**
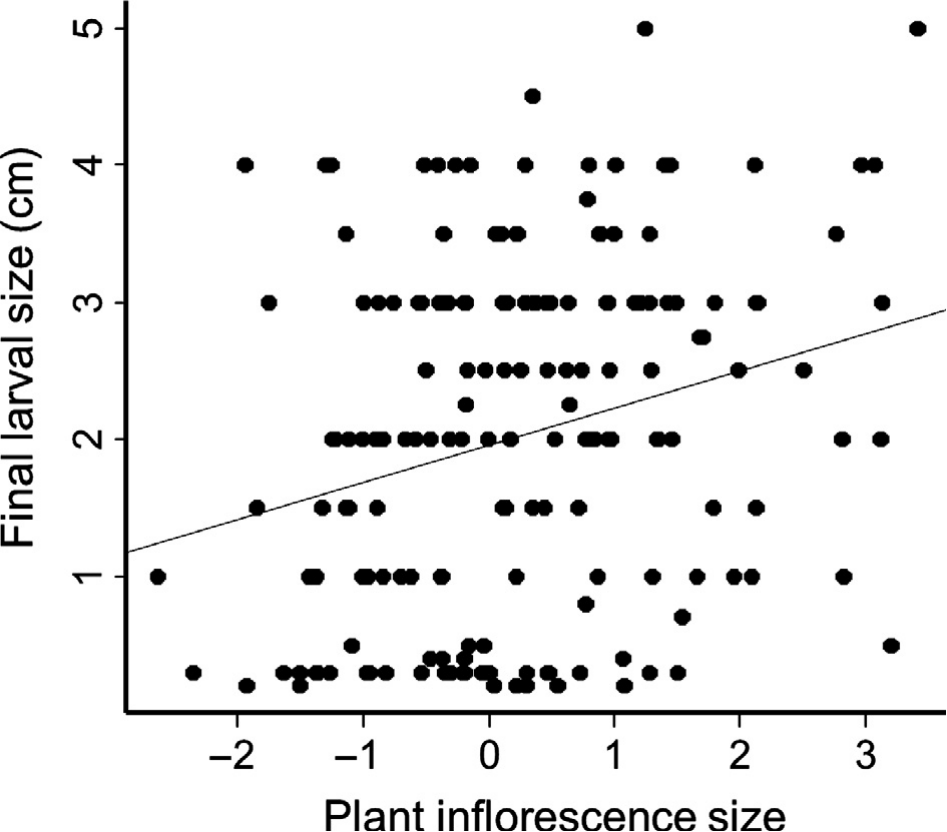
Relationship between final larval size in cm and plant inflorescence size, *n* = 173, *P* = 0.0019, *t* = 3.16, *r* ^2^ = 0.067. Plant inflorescence size represents the first principal component between log_10_‐transformed flower shoot volume and square root‐transformed number of flowers.

Larval size was positively correlated with plant inflorescence size (*P = *0.0019, *t*‐value = 3.16, Fig. 1), but not with flower size (*P *=* *0.77, *t*‐value = 0.30) or flowering phenology (*P *=* *0.94, *t*‐value = 0.07, Table S2‐3).

**Figure 2:**
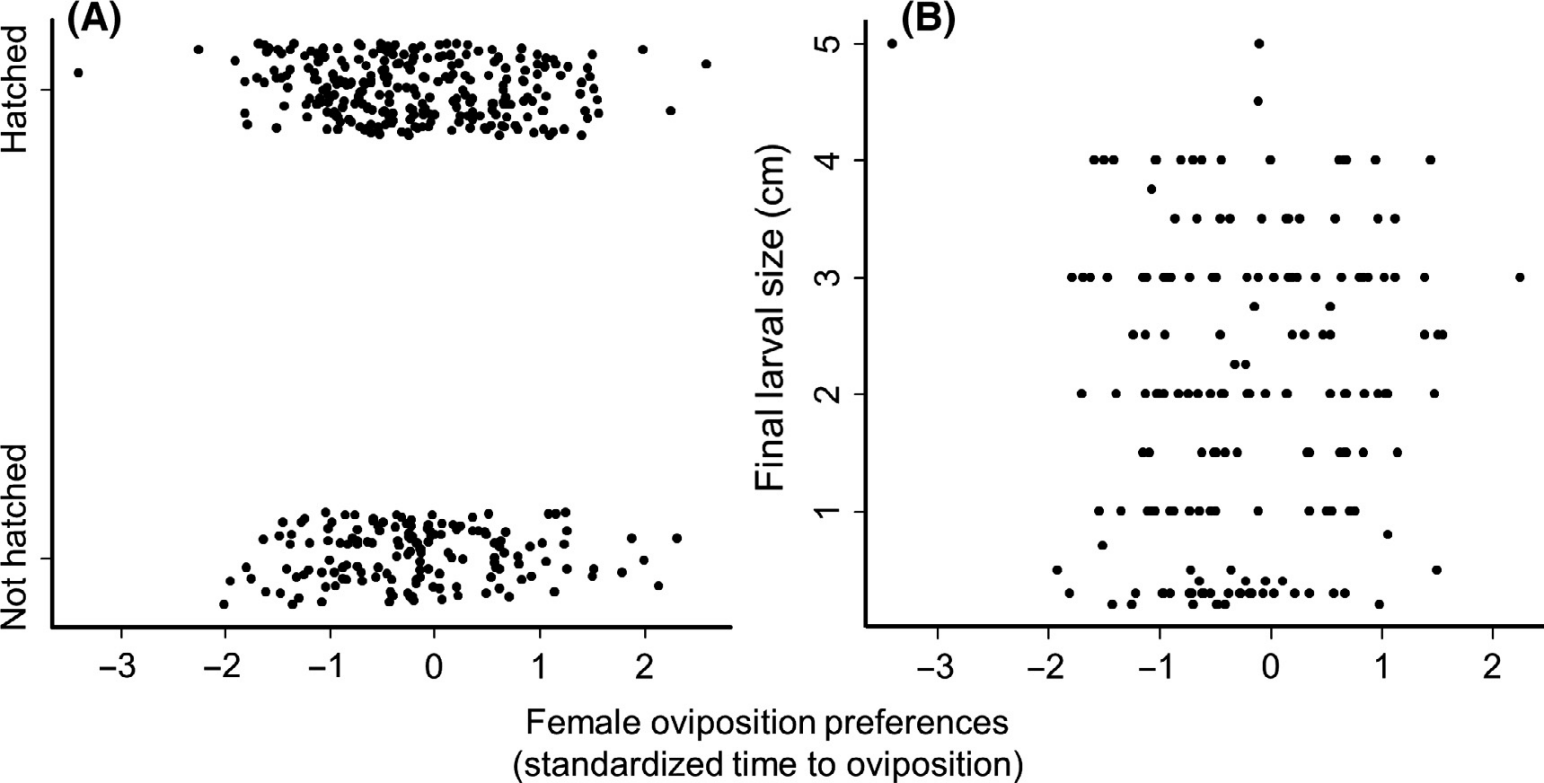
Relationship between female oviposition preference under controlled conditions and (A) the probability of eggs hatching, *n* = 419, *P* = 0.73, *z* = −0.35, (B) final larval size in cm, *n* = 173, *P* = 0.85, *t* = −0.19. Female oviposition preference was estimated in a cage experiment, and standardized within trials by subtracting the mean oviposition time within each experimental trial from the individual measure, and dividing this difference by the standard deviation of the trial. Negative oviposition preference values correspond to that a plant was more preferred compared to other plants and positive values correspond to that a plant was less preferred.

Butterfly female oviposition preference was neither correlated with egg survival (*P = *0.73, *z*‐value = −0.35, Fig. 2A, Table S2‐4) nor larval performance (*P = *0.85, *t*‐value = −0.19, Fig. 2B, Table S2‐5). Moreover, larvae which were added to plants rejected for oviposition during the cage experiment grew equally well as larvae on plants that were oviposited upon (mean larval length_oviposited_ = 2.03 cm, SD_oviposited_ = 1.27, mean larval length_added_ = 1.49 cm, SD_added_ = 1.11, *P = *0.28, *t*‐value = 1.09, Table S2‐6).

Larval performance under experimental conditions was not correlated with the proportion of plants within the corresponding population that were oviposited upon in the field (*r* ^2^ = 0.004, F_1, 17_ = 0.08, *P *=* *0.79, Table S2‐7).

## Discussion

We found no effect of ploidy type or population of origin on egg survival or larval performance. The only investigated plant trait which affected larval development was plant inflorescence size; increased plant inflorescence size allowed the larvae to reach a larger final size. Female oviposition preference under controlled conditions was not correlated with larval performance, and larvae grew equally well on plants rejected or chosen for oviposition. Lastly, among‐population variation in larval performance was not correlated with the proportion of plants that were oviposited upon in the field.

Plant ploidy level has been shown to affect oviposition preferences of insect herbivores in several previous studies (Janz and Thompson 2002; Halverson et al. 2008). Such differences are believed to be due to differences in plant morphology (Janz and Thompson 2002; Halverson et al. 2008). However, no study has previously investigated if such differences in preference correspond to differences in offspring development. Although *A. cardamines* females show a preference for allopatric tetraploid populations, they are known to prefer octoploid individuals in sympatric populations (Arvanitis et al. 2008). In this study, however, there was no evidence for an increased final larval size when larvae fed on octoploids compared to feeding on tetraploids, or for differences in the hatching frequency of *A. cardamines* eggs between the ploidy levels. Moreover, the population of origin of the plants did not affect egg survival or final larval size, indicating that there are no genetic differences in plant quality as food for the butterfly larvae between populations. Overall, our results show that the observed differences in host plant utilization by *A. cardamines* among the two ploidy types are not associated with differences in egg survival or larval development.

Locating a suitable host plant individual is crucial for the ovipositing female butterfly, and *A. cardamines* mainly uses visual cues when locating host plants (Wiklund and Åhrberg 1978; Courtney 1982). In our study, inflorescence size, but not flower size, had an effect on final larval size. That the size of the host plant affected larval growth was not surprising, since studies in other butterfly systems have shown that larger quantities of food normally result in increased final larval size (Boggs and Freeman 2005). However, the lack of correlation between flower size and final larval size was unexpected as a previous study showed that female *A. cardamines* under controlled conditions uses both traits as a cue to decide where to oviposit (König et al. 2014b). Plant inflorescence size is first evaluated after the female has located and landed on the inflorescence (Wiklund and Åhrberg 1978). Flower size may therefore not be the best predictor of an optimal larval food source but rather a way for females to locate a host plant.

Our results suggest that the criteria used by the female to decide where to oviposit, are not correlated with the future fitness of individual larvae. There are several possible explanations for such a lack of correlation. First, the fact that *A. cardamines* lays eggs on virtually all brassicaeceaous plants they encounter (Wiklund and Friberg 2009) may limit the female's ability to evaluate the quality of individual plants. Second, the survival rate for larvae in the field on *C. pratensis* is highly variable among years (Wiklund and Friberg 2009). Third, female *A. cardamines* searching for host plants might be time limited since they are able to produce as many as 170 eggs (Wiklund et al. 2001) and only oviposit one egg per host plant. Courtney and Duggan (1983) considered “egg shortfall” to be the major determinant of population size of British *A. cardamines*. If accepting only plants allowing maximal larval development leads to a reduced total number of eggs laid, then maximizing individual larval fitness might result in a lower female total fitness (Rosenheim et al. 2008).

In our study, larval performance under experimental conditions was not correlated with the proportion of plants within the corresponding population that were oviposited upon in the field. However, it is not obvious what kind of relationship between oviposition frequencies in the field and larval performance that would be expected based on theory. If attack rates are not primarily determined by host plant quality, then plant populations exposed to sustained high intensities of attacks should be selected for increased defense levels against larval herbivory, and thus lead to poorer larval performance. Previous studies have indeed found that butterfly attacks have important effects on *C. pratensis'* fitness (Arvanitis et al. 2007; König et al. 2014a). On the other hand, if attack rates respond strongly to defense level, then herbivores should be expected to be more abundant in populations with lower levels of defense. The fact that we found no evidence of increased defenses against the larvae within preferred populations, or a tendency by the female to locate the populations with lower defense levels for oviposition, thus suggest either that none of the above mentioned mechanisms are important in this system or that they are balancing each other. It is also possible that observed oviposition patterns in natural populations are the result of habitat differences among populations in combination with butterfly preferences for certain habitats, rather than an active choice based on host plant quality by the female. Habitat differences, e.g., in terms of canopy cover, have been shown to be the main cause of differences in the proportion of host plants used for oviposition among both tetraploid and octoploid populations of *C. pratensis* (Arvanitis et al. 2007, 2008). However, it is unknown if such habitat preferences by *A. cardamines* are associated with increased larval or female survival. Still, it seems likely that open habitats favor both females, whose flight is dependent on solar radiation and temperature (Courtney and Duggan 1983), and larvae, whose growth rate increases with temperature (Bryant et al. 2003). If larvae grow faster in the habitats where tetraploids grow compared to octoploid habitats, then female *A. cardamines* oviposition choices are somewhat in line with the “mother knows best” hypothesis even though the tetraploids do not provide the best food source with regard to the amount of plant material. This kind of differences could not be evaluated in our experiment since all plants were kept under the same light and temperature conditions.

In a world full of nonhost plants, the most important part of oviposition is to locate the potential host plants. In our study system, *A. cardamines* is highly dependent on visual cues for the initial host recognition (Wiklund and Åhrberg 1978; Courtney 1982), and the female is only able after close range examination to evaluate the quality of the located plant. The fact that *A. cardamines* does not seem to be a strong case of mother knows best, might thus be the result of females maximizing their own fitness by reducing search time and using hosts of a sufficient quality rather than aiming for the best host. The costs of using suboptimal hosts for oviposition might also be partly offset by the mobility of later instar larvae. We interpret that the lack of positive correlation between oviposition preference in the field and larval performance can be explained by the fact that female oviposition behavior and host choice is being selected to maximize the total fitness of the females rather than that of their individual offspring.

## Conflict of Interest

None declared.

## Supporting information


**Appendix S1.** Data used for the models.
**Table S1‐1.** Data for the enclosed preference/performance experiments.
**Table S1‐2.** Data used to compare larvae growing on plants chosen for oviposition and plants rejected for oviposition.
**Table S1‐3.** Data for comparision between performance under controlled conditions and oviposition preferences in field populations under natural conditions.

**Appendix S2.** Models and result tables for ordinary generalized linear models, and generalized linear mixed effects models analyzed in R 3.2.3.
**Table S2‐1.** Egg survival by *Anthocharis cardamines* in two ploidy types and 51 populations of *Cardamine pratensis*.
**Table S2‐2.** Larval developments by *A. cardamines* in two ploidy types and 30 populations of *C. pratensis*.
**Table S2‐3.** The effect of individual plant traits of two ploidy types of *C. pratensis* on the development of *A. cardamines* larvae.
**Table S2‐4.** The effect of oviposition preferences by female *A. cardamines* on egg survival on two ploidy types of *C. pratensis* under experimental conditions.
**Table S2‐5.** The effect oviposition preferences by female *A. cardamines* on larval development on two ploidy types of *C. pratensis* under experimental conditions.
**Table S2‐6.** Comparison of *A. cardamines* larval development between larvae feeding on plants chosen for oviposition and larvae feeding on plants rejected for oviposition by butterfly females.
**Table S2‐7.** Correlation between *A. cardamines* larval performance under experimental conditions and female host plant use in the field for two ploidy types of *C. pratensis*.
