# Supplementary material for: Butterfly oviposition preference is not related to larval performance on a polyploid herb
Source: Ecol Evol. 2016 Mar 20;6(9):2781–9. doi: 10.1002/ece3.2067 (PMC4863005; doi:10.1002/ece3.2067)
Supplement: Supplementary file 1 — Appendix S1. Data used for the models. Table S1‐1. Data for the enclosed preference/performance experiments. Table S1‐2. Data used to compare larvae growing on plants chosen for oviposition and plants rejected for oviposition. Table S1‐3. Data for comparision between performance under controlled conditions and oviposition preferences in field populations under natural conditions. [file ECE3-6-2781-s001.docx]

**Supplementary Material Appendix 1**

Butterfly oviposition preference is not related to larval performance on a polyploid herb

**Malin A. E. König^1^, Christer Wiklund^2^, Johan Ehrlén^1^**

^1^Department of Ecology, Environment and Plant Sciences, Stockholm University, SE106 91 Stockholm, Sweden

^2^Department of Zoology, Stockholm University, SE106 91 Stockholm, Sweden 6

**Table of contents**

**Table S1- 1-3.** The complete dataset for all models used in the papper.

**Table S1-1.** The ploidy type, plant population, plant genet, flowering phenology (number of days since the first open flower), inflorescence size (the first principle component between number of flowers and flower shoot size) and flower diameter of each ramet of *C. pratensis*. Also the data for if the eggs of *A. cardamines* hatched or not (1 or 0) and the final larval size (cm). NA in final larval size are found for eggs that did not hatch and plant genets that are represented by less than two hatched larva and thus not included in our statistical analyses.

| Ploidy type | Plant population | Plant genet | Ramet | Butterfly female | Hatched or not hatched | Oviposition preferences under controlled conditions | Phenology | Inflorescence size | Flower diameter | Final larval size |
| --- | --- | --- | --- | --- | --- | --- | --- | --- | --- | --- |
| Octoploid | Almvik | 1a | 1A | E- | 1 | -0.59019 | 5 | -0.94503 | 16.5 | 0.3 |
| Octoploid | Almvik | 1a | 2 | S7 | 1 | -0.86112 | 5 | -1.14237 | 18.5 | 3.5 |
| Octoploid | Almvik | 1a | 3 | E10 | 0 | -0.25115 | 11 | -1.03012 | 18 | NA |
| Octoploid | Almvik | 2a | 1 | E4 | 0 | -0.44203 | 0 | -1.0079 | 14 | NA |
| Octoploid | Almvik | 2a | 2 | V | 1 | -0.10074 | 6 | 1.243931 | 22.5 | 5 |
| Octoploid | Almvik | 2a | 3 | S7 | 1 | 0.358084 | 5 | -0.81816 | 19 | 0.3 |
| Octoploid | Almvik | 3a | 1 | S2 | 1 | -1.24361 | 3 | 0.305476 | 21 | 0.2 |
| Octoploid | Almvik | 3a | 2B | E8 | 0 | -0.14529 | 4 | -0.95729 | 19 | NA |
| Octoploid | Almvik | 3a | 2A | E- | 1 | -0.51865 | 5 | -0.18089 | 18 | 3 |
| Octoploid | Aspa 2 | 2b | 1 | E7 | 1 | -0.21841 | 5 | -0.17938 | 19 | 2.25 |
| Octoploid | Aspa 2 | 2b | 2 | E13 | 0 | -0.52873 | 7 | 0.549769 | 19 | NA |
| Octoploid | Aspa 2 | 2b | 3 | S8 | 1 | -0.72457 | 9 | -2.35068 | 20 | 0.3 |
| Octoploid | Aspa 2 | 3b | 1 | E5 | 1 | 0.358474 | 3 | 1.299654 | 21 | 1 |
| Octoploid | Aspa 2 | 3b | 2 | S3 | 1 | -0.65704 | 3 | 0.885717 | 23.5 | 3.5 |
| Octoploid | Aspa 2 | 4b | 1 | E2 | 1 | -0.88788 | 0 | 1.283588 | 23 | 3 |
| Octoploid | Aspa 2 | 4b | 2 | S1 | 0 | -1.64308 | 3 | 1.162791 | 26 | NA |
| Octoploid | Aspa 2 | 4b | 3 | E6 | 1 | -0.34853 | 3 | -0.04761 | 21 | 0.5 |
| Tetraploid | Bogslund | 1d | 1 | E4 | 0 | 0.417176 | 0 | -1.0488 | 10 | NA |
| Tetraploid | Bogslund | 1d | 3 | E7 | 1 | 0.862154 | 4 | -0.45341 | 14 | NA |
| Tetraploid | Bogslund | 2d | 1 | E1 | 1 | 1.061899 | 1 | 0.768081 | 12.5 | 0.8 |
| Tetraploid | Bogslund | 2d | 2 | S1 | 0 | -0.87664 | 3 | 0.516219 | 14 | NA |
| Tetraploid | Bogslund | 2d | 3 | S5 | 1 | 0.977324 | 3 | 0.095308 | 12.75 | 3.5 |
| Tetraploid | Bogslund | 3d | 1 | S2 | 1 | 0.3285 | 3 | -1.33163 | 15 | 1.5 |
| Tetraploid | Bogslund | 3d | 2 | S3 | 1 | -1.42136 | 2 | 0.216749 | 15 | 0.2 |
| Tetraploid | Bogslund | 3d | 3 | E- | 1 | 0.97921 | 3 | -1.92895 | 12.5 | 0.2 |
| Tetraploid | Bogslund | 4d | 2 | E3 | 1 | 1.981232 | 0 | -0.46096 | 11.5 | NA |
| Tetraploid | Bogslund | 4d | 3A | E- | 0 | -0.85776 | 3 | 0.116581 | 12.25 | NA |
| Octoploid | Bysjön | 1g | 1 | E1 | 0 | -0.74251 | 3 | 1.714972 | 21.5 | NA |
| Octoploid | Bysjön | 1g | 2 | E3 | 0 | -0.00473 | 0 | 0.728116 | 20 | NA |
| Octoploid | Bysjön | 1g | 3 | E1 | 1 | -0.09374 | 4 | 0.601385 | 20 | NA |
| Octoploid | Bysjön | 2g | 1 | S2 | 1 | -0.71942 | 5 | -0.16257 | 21 | 0.5 |
| Octoploid | Bysjön | 2g | 2 | S8 | 1 | -0.0595 | 4 | -1.6399 | 19 | 0.3 |
| Octoploid | Bysjön | 2g | 2B | S8 | 0 | -0.69841 | 4 | 0.10858 | 19 | NA |
| Octoploid | Bysjön | 3g | 1 | E5 | 1 | -0.23702 | 4 | 0.564125 | 22 | NA |
| Octoploid | Bysjön | 3g | 2 | E3 | 0 | -1.32875 | 3 | 0.008183 | 21 | NA |
| Octoploid | Bysjön | 3g | 3 | S2 | 0 | -1.01762 | 4 | 1.226053 | 23.5 | NA |
| Octoploid | Bysjön | 4g | 1 | E4 | 0 | -0.14597 | 4 | 1.003021 | 21 | NA |
| Octoploid | Bysjön | 4g | 2A | V | 1 | 0.675153 | 6 | -0.05383 | 22 | 0.3 |
| Octoploid | Bysjön | 4g | 2B | E7 | 1 | 0.680789 | 8 | -1.25374 | 19 | 4 |
| Octoploid | Bysjön | 4g | 3 | E7 | 1 | -0.03871 | 5 | -0.19245 | 20.75 | 0.4 |
| Octoploid | Bölsäter 1 | 2h | 1 | S2 | 1 | -1.80997 | 2 | -0.53817 | 22.5 | 0.3 |
| Octoploid | Bölsäter 1 | 2h | 1B | E7 | 1 | 0.666701 | 4 | -1.93766 | 21 | 4 |
| Octoploid | Bölsäter 1 | 2h | 2B | E8 | 0 | -0.182 | 5 | 0.073182 | 22.5 | NA |
| Octoploid | Bölsäter 1 | 3h | 1 | E8 | 0 | -0.02616 | 3 | -0.28844 | 19.5 | NA |
| Octoploid | Bölsäter 1 | 3h | 2 | E11 | 1 | 0.615346 | 7 | -0.51403 | 19.5 | 4 |
| Octoploid | Bölsäter 1 | 3h | 3 | S8 | 1 | -1.53598 | 8 | -2.63969 | 18 | 1 |
| Octoploid | Bölsäter 1 | 4h | 1 | E2 | 1 | 0.202875 | 0 | -0.02583 | 20 | 2.5 |
| Octoploid | Bölsäter 1 | 4h | 2 | E4 | 1 | -0.08018 | 4 | 0.221477 | 20 | 3.5 |
| Octoploid | Bölsäter 1 | 4h | 3 | E8 | 0 | 1.258237 | 2 | -2.65388 | 21.5 | NA |
| Tetraploid | Bölsäter 2 | 1i | 1 | S2 | 0 | -0.01566 | 4 | 0.321984 | 13.25 | NA |
| Tetraploid | Bölsäter 2 | 1i | 2 | E6 | 1 | -0.87963 | 3 | -0.4308 | 13 | NA |
| Tetraploid | Bölsäter 2 | 3i | 1 | E1 | 0 | 0.903849 | 2 | 2.319197 | 13.5 | NA |
| Tetraploid | Bölsäter 2 | 3i | 2 | E5 | 0 | -1.08326 | 4 | 2.040715 | 14.75 | NA |
| Tetraploid | Bölsäter 3 | 3j | 1 | E2 | 1 | -0.45421 | 2 | 1.134961 | 15.25 | NA |
| Tetraploid | Bölsäter 3 | 3j | 2 | S5 | 1 | 1.048188 | 0 | -2.38856 | 12.5 | NA |
| Tetraploid | Bölsäter 3 | 3j | 3 | S3 | 1 | 0.602388 | 8 | -0.52169 | 13.25 | NA |
| Tetraploid | Bölsäter 3 | 4j | 1 | E1 | 0 | -0.53815 | 4 | 1.251608 | 14.75 | NA |
| Tetraploid | Bölsäter 3 | 4j | 2 | S2 | 1 | -0.25811 | 2 | -1.54843 | 15 | NA |
| Tetraploid | Dagnäs 1 | 1q | 1 | E3 | 1 | 0.543578 | 2 | 1.706693 | 16.5 | 2.75 |
| Tetraploid | Dagnäs 1 | 1q | 2 | S2 | 1 | -1.23233 | 4 | 0.74049 | 14 | 2.5 |
| Tetraploid | Dagnäs 1 | 1q | 3 | S8 | 1 | -0.13753 | 4 | 1.677299 | 16.25 | 2.75 |
| Tetraploid | Dagnäs 1 | 2q | 1 | V | 1 | 1.555846 | 1 | 1.290266 | 17 | 2.5 |
| Tetraploid | Dagnäs 1 | 2q | 2 | E8 | 1 | -0.499 | 2 | 2.095826 | 13.25 | 1 |
| Tetraploid | Dagnäs 1 | 2q | 3 | E13 | 0 | -2.01132 | 3 | 1.113168 | 15.5 | NA |
| Tetraploid | Dagnäs 1 | 3q | 1 | E7 | 1 | -1.91191 | 3 | 3.205905 | 15.75 | 0.5 |
| Tetraploid | Dagnäs 1 | 3q | 2 | S7 | 1 | -0.43674 | 3 | -0.15324 | 16 | 4 |
| Tetraploid | Dagnäs 1 | 3q | 3 | E8 | 1 | -0.48664 | 4 | 0.546581 | 17 | 0.2 |
| Tetraploid | Dagnäs 1 | 4q | 1 | E4 | 0 | -0.54072 | 1 | 1.300316 | 16.25 | NA |
| Tetraploid | Dagnäs 1 | 4q | 2A | E- | 1 | 0.411344 | 3 | -0.56105 | 14 | 3 |
| Tetraploid | Dagnäs 1 | 4q | 3 | E10 | 1 | -0.21394 | 7 | -0.37337 | 13.5 | 0.4 |
| Tetraploid | Dagnäs 2 | 1r | 1 | E8 | 0 | -0.40929 | 2 | 2.237206 | 14.5 | NA |
| Tetraploid | Dagnäs 2 | 1r | 3 | E10 | 0 | 0.798097 | 6 | 0.549529 | 16.25 | NA |
| Tetraploid | Dagnäs 2 | 2r | 1A | E- | 1 | -0.68293 | 3 | 0.045693 | 12 | NA |
| Tetraploid | Dagnäs 2 | 2r | 2 | E8 | 0 | -0.238 | 3 | 0.284249 | 16.5 | NA |
| Tetraploid | Dagnäs 2 | 3r | 1 | E1 | 1 | -0.51963 | 2 | 2.111971 | 12.83333 | NA |
| Tetraploid | Dagnäs 2 | 3r | 2 | E5 | 1 | 0.233106 | 2 | 1.968879 | 15.75 | NA |
| Tetraploid | Dagnäs 2 | 3r | 3 | S8 | 1 | -0.07281 | 3 | 0.328703 | 17 | NA |
| Octoploid | Dammen | 1s | 1 | S2 | 0 | -1.25618 | 2 | 0.229933 | 20.5 | NA |
| Octoploid | Dammen | 1s | 2 | V | 1 | -0.64182 | 3 | -0.46842 | 17.5 | 0.4 |
| Octoploid | Dammen | 1s | 3 | E10 | 1 | 0.000768 | 10 | -1.30472 | 18.5 | 4 |
| Octoploid | Dammen | 2s | 2 | V | 1 | -0.5285 | 5 | 1.851308 | 21 | NA |
| Octoploid | Dammen | 2s | 3 | E8 | 0 | -0.82952 | 5 | 1.199058 | 20.5 | NA |
| Octoploid | Dammen | 3s | 1 | E8 | 1 | 0.039247 | 4 | -0.2023 | 19.5 | 0.3 |
| Octoploid | Dammen | 3s | 2A | E- | 1 | -0.44711 | 4 | 0.053583 | 20 | 3.5 |
| Octoploid | Dammen | 3s | 3 | E10 | 0 | 0.676936 | 8 | -1.52747 | 20.5 | NA |
| Octoploid | Dammen | 4s | 1 | E7 | 0 | -0.25929 | 7 | 0.628144 | 18 | NA |
| Octoploid | Dammen | 4s | 2 | E7 | 1 | 0.843386 | 3 | -0.99434 | 18 | 3 |
| Octoploid | Dammen | 4s | 3 | S7 | 1 | -0.20492 | 5 | -0.19353 | 20 | 3 |
| Octoploid | Davik | 1t | 1 | V | 1 | -0.60186 | 3 | -0.45585 | 21.5 | NA |
| Octoploid | Davik | 1t | 2 | E10 | 0 | 1.061581 | 9 | -2.01316 | 22.5 | NA |
| Octoploid | Davik | 4t | 1 | E7 | 1 | -0.20244 | 5 | -0.73777 | 21.5 | NA |
| Octoploid | Davik | 4t | 2 | E8 | 1 | -1.19829 | 5 | -1.72135 | 21 | NA |
| Octoploid | Davik | 4t | 3 | E10 | 0 | -0.36277 | 11 | -0.41887 | 23.5 | NA |
| Octoploid | Djupbrodal | 1u | 1 | E2 | 1 | 0.294866 | 3 | 0.816899 | 25 | NA |
| Octoploid | Djupbrodal | 1u | 2 | E3 | 0 | -0.21751 | 1 | -1.24744 | 21.5 | NA |
| Octoploid | Djupbrodal | 1u | 3 | E1 | 0 | -0.14929 | 2 | 1.508863 | 23 | NA |
| Octoploid | Djupbrodal | 2u | 1 | E1 | 0 | -0.78202 | 4 | 0.872187 | 23 | NA |
| Octoploid | Djupbrodal | 2u | 2 | E4 | 0 | -1.039 | 4 | 0.479234 | 22 | NA |
| Octoploid | Djupbrodal | 2u | 3 | E5 | 1 | 1.455445 | 4 | -0.7487 | 23.5 | NA |
| Octoploid | Djupbrodal | 3u | 1 | E2 | 0 | -1.45298 | 3 | 0.252785 | 23.5 | NA |
| Octoploid | Djupbrodal | 3u | 2 | S5 | 1 | -0.97076 | 4 | 0.035002 | 22.5 | NA |
| Octoploid | Djupbrodal | 3u | 3 | E4 | 1 | 2.584336 | 4 | -2.17062 | 16.5 | NA |
| Tetraploid | Edeby 1 | 1w | 1 | E3 | 1 | 0.846398 | 0 | 0.972515 | 14.25 | 2 |
| Tetraploid | Edeby 1 | 1w | 2 | S5 | 1 | 1.024567 | 3 | 0.932475 | 14.25 | 3 |
| Tetraploid | Edeby 1 | 1w | 3 | S2 | 0 | -0.24229 | 4 | 0.860485 | 13.75 | NA |
| Tetraploid | Edeby 1 | 2w | 1 | E3 | 1 | 1.389143 | 3 | 1.986447 | 11.875 | 2.5 |
| Tetraploid | Edeby 1 | 2w | 2 | S5 | 1 | 1.147257 | 0 | 0.438697 | 14.5 | 1.5 |
| Tetraploid | Edeby 1 | 2w | 3 | E7 | 0 | 0.786111 | 1 | -0.97886 | 11.25 | NA |
| Tetraploid | Edeby 1 | 3w | 1 | E4 | 1 | 2.24724 | 0 | 1.487197 | 11 | 3 |
| Tetraploid | Edeby 1 | 3w | 3 | S2 | 1 | 0.16425 | 3 | 1.280379 | 17 | 3.5 |
| Tetraploid | Edeby 1 | 3w | 3B | S2 | 1 | 0.558365 | 7 | -1.43789 | 13 | 1 |
| Octoploid | Edeby 2 | 1x | 1 | V | 1 | -1.38489 | 1 | 1.350663 | 22.5 | 2 |
| Octoploid | Edeby 2 | 1x | 2 | E7 | 1 | -0.48914 | 3 | 1.796949 | 21.5 | 3 |
| Octoploid | Edeby 2 | 2x | 1 | E3 | 0 | -0.84557 | 4 | 3.188627 | 21 | NA |
| Octoploid | Edeby 2 | 2x | 2 | S5 | 1 | -0.79426 | 0 | 4.689117 | 22.5 | NA |
| Octoploid | Edeby 2 | 3x | 1 | S2 | 1 | 0.545835 | 7 | 1.653903 | 20 | 1 |
| Octoploid | Edeby 2 | 3x | 2 | S8 | 1 | 0.810884 | 4 | 2.143588 | 19.5 | 3 |
| Octoploid | Edeby 2 | 3x | 3 | E7 | 1 | -0.20897 | 3 | 0.840545 | 16.5 | 2 |
| Octoploid | Edeby 2 | 4x | 1 | S2 | 0 | -1.00685 | 0 | 0.380874 | 15.5 | NA |
| Octoploid | Edeby 2 | 4x | 2 | E8 | 1 | 0.117874 | 3 | 1.067094 | 20.5 | 0.4 |
| Octoploid | Edeby 2 | 4x | 3 | S8 | 1 | -0.41797 | 5 | 0.524457 | 20.5 | 2 |
| Tetraploid | Gravfältet | 1aa | 1 | S4 | 1 | -0.10495 | 3 | 0.861116 | 9 | 1 |
| Tetraploid | Gravfältet | 1aa | 2 | E7 | 1 | 0.680789 | 7 | -0.89079 | 7.5 | 1.5 |
| Tetraploid | Gravfältet | 1aa | 3 | E8 | 0 | 0.161734 | 4 | -0.41675 | 13.5 | NA |
| Tetraploid | Gravfältet | 2aa | 1 | E7 | 1 | 0.167739 | 1 | -0.3918 | 14.5 | 3 |
| Tetraploid | Gravfältet | 2aa | 3A | E- | 1 | 0.840573 | 4 | 0.344359 | 13.5 | 1.5 |
| Tetraploid | Gravfältet | 3aa | 1 | S2 | 1 | 0.683664 | 7 | -1.1163 | 10.5 | 2 |
| Tetraploid | Gravfältet | 3aa | 2 | S5 | 1 | -1.69871 | 3 | 3.119978 | 15.25 | 2 |
| Tetraploid | Gravfältet | 3aa | 3 | E6 | 1 | -0.7203 | 1 | -0.8461 | 9 | 1 |
| Tetraploid | Grinda | 1ab | 1 | E3 | 1 | -1.02676 | 1 | 1.425547 | 17 | NA |
| Tetraploid | Grinda | 1ab | 2 | E1 | 1 | -1.1307 | 2 | 4.241095 | 15.5 | NA |
| Tetraploid | Grinda | 1ab | 3 | E7 | 1 | 0.314902 | 3 | 1.309463 | 16 | NA |
| Tetraploid | Grinda | 2ab | 1 | S7 | 1 | 0.291849 | 3 | -0.31874 | 14.5 | NA |
| Tetraploid | Grinda | 2ab | 2 | E8 | 0 | -0.27733 | 3 | -1.35746 | 15 | NA |
| Octoploid | Gustav | 1ad | 1 | E1 | 0 | -0.22336 | 3 | 0.215858 | 25.5 | NA |
| Octoploid | Gustav | 1ad | 2 | S4 | 0 | -0.04001 | 3 | -1.67098 | 25 | NA |
| Octoploid | Gustav | 1ad | 3 | V | 0 | 0.03746 | 3 | -1.70627 | 23 | NA |
| Octoploid | Gustav | 2ad | 2 | E3 | 0 | -0.96225 | 0 | 0.277441 | 21 | NA |
| Octoploid | Gustav | 2ad | 3B | E10 | 1 | 0.97796 | 13 | -1.26514 | 25.5 | NA |
| Octoploid | Gustav | 3ad | 1 | E3 | 0 | -0.42278 | 3 | -1.30824 | 29 | NA |
| Octoploid | Gustav | 3ad | 2 | S3 | 0 | -1.37124 | 4 | -1.45795 | 23 | NA |
| Octoploid | Gustav | 4ad | 1A | S2 | 1 | -0.25422 | 4 | -0.66842 | 28 | NA |
| Octoploid | Gustav | 4ad | 2 | E6 | 1 | -0.50786 | 4 | -1.83624 | 21.5 | NA |
| Octoploid | Horssjön | 1ae | 1 | S2 | 1 | -0.39889 | 3 | 0.558411 | 23.5 | NA |
| Octoploid | Horssjön | 1ae | 3 | E7 | 0 | -0.13667 | 9 | -0.28713 | 20 | NA |
| Octoploid | Horssjön | 2ae | 1 | E1 | 0 | -1.01909 | 3 | 1.121263 | 19 | NA |
| Octoploid | Horssjön | 2ae | 2 | E3 | 0 | -0.36239 | 4 | 0.923694 | 16 | NA |
| Octoploid | Horssjön | 2ae | 3 | S3 | 0 | 0.574095 | 8 | -0.30506 | 19.5 | NA |
| Octoploid | Horssjön | 3ae | 1 | E4 | 0 | -0.17887 | 3 | 0.508773 | 12.5 | NA |
| Octoploid | Horssjön | 3ae | 2 | S4 | 1 | -1.06056 | 4 | 1.118119 | 23 | NA |
| Octoploid | Horssjön | 4ae | 1 | E4 | 1 | -1.51128 | 0 | -0.12546 | 18 | NA |
| Octoploid | Horssjön | 4ae | 2 | E5 | 1 | 1.267393 | 4 | 0.634109 | 20.5 | NA |
| Octoploid | Inskogsbergen | 1ah | 1 | E4 | 1 | -0.90256 | 2 | -1.36738 | 20.5 | 0.3 |
| Octoploid | Inskogsbergen | 1ah | 2 | E8 | 1 | 0.644195 | 3 | -1.75381 | 20 | 3 |
| Octoploid | Inskogsbergen | 1ah | 3 | S8 | 1 | -1.022 | 5 | -0.27025 | 20 | 4 |
| Octoploid | Inskogsbergen | 3ah | 1 | S1 | 0 | -1.95533 | 3 | 1.628452 | 21 | NA |
| Octoploid | Inskogsbergen | 3ah | 2 | S3 | 0 | 0.479788 | 6 | -0.04136 | 22.25 | NA |
| Octoploid | Inskogsbergen | 3ah | 3 | S7 | 1 | 0.339707 | 5 | -1.1173 | 17.5 | NA |
| Octoploid | Inskogsbergen | 4ah | 1 | S3 | 1 | -1.49654 | 0 | 3.070148 | 21.5 | 4 |
| Octoploid | Inskogsbergen | 4ah | 2 | S8 | 0 | -1.03868 | 4 | 0.594967 | 17 | NA |
| Octoploid | Inskogsbergen | 4ah | 3 | E7 | 1 | 0.945323 | 4 | 0.290335 | 15.25 | 4 |
| Tetraploid | Kallmyra 1 | 1aj | 1 | E5 | 1 | -0.86386 | 4 | 1.945529 | 16.5 | 1 |
| Tetraploid | Kallmyra 1 | 1aj | 3 | V | 1 | -0.32216 | 1 | 0.639062 | 15.16667 | 2.25 |
| Tetraploid | Kallmyra 1 | 3aj | 1 | E3 | 0 | 0.066199 | 0 | 0.135708 | 12 | NA |
| Tetraploid | Kallmyra 1 | 3aj | 3 | S2 | 1 | 0.140786 | 3 | 1.420953 | 15.5 | NA |
| Tetraploid | Kallmyra 1 | 4aj | 1 | S7 | 1 | -0.27115 | 2 | -0.35359 | 10.5 | 0.3 |
| Tetraploid | Kallmyra 1 | 4aj | 2 | E8 | 0 | -0.57036 | 3 | 0.736994 | 13 | NA |
| Tetraploid | Kallmyra 1 | 4aj | 3 | S7 | 1 | -0.63991 | 2 | -1.37289 | 14 | 1 |
| Tetraploid | Kallmyra 2 | 1ak | 1 | S8 | 1 | -0.26696 | 3 | 0.72059 | 13.75 | 0.3 |
| Tetraploid | Kallmyra 2 | 1ak | 2 | E10 | 1 | 0.511604 | 2 | -0.96625 | 12 | 1 |
| Tetraploid | Kallmyra 2 | 2ak | 1 | E8 | 0 | -0.31958 | 2 | -0.83218 | 16 | NA |
| Tetraploid | Kallmyra 2 | 2ak | 2 | S7 | 1 | -0.95592 | 3 | 0.460067 | 13.75 | 0.3 |
| Tetraploid | Kallmyra 2 | 2ak | 3 | E11 | 1 | 0.715301 | 5 | -0.36655 | 13 | 1 |
| Tetraploid | Kallmyra 2 | 3ak | 1 | S2 | 1 | -1.14883 | 3 | 1.163046 | 14.25 | 3 |
| Tetraploid | Kallmyra 2 | 3ak | 2 | V | 1 | 1.396016 | 3 | 1.204658 | 16 | 3 |
| Tetraploid | Kallmyra 2 | 3ak | 3 | E13 | 0 | -1.6109 | 7 | -0.5514 | 11.7 | NA |
| Tetraploid | Kallmyra 2 | 4ak | 1 | S2 | 0 | -1.36117 | 0 | 0.866816 | 15 | NA |
| Tetraploid | Kallmyra 2 | 4ak | 2A | S8 | 1 | -0.0459 | 2 | 0.858735 | 13.25 | NA |
| Tetraploid | Kallmyra 3 | 1al | 1 | E4 | 1 | 0.479038 | 1 | 0.123878 | 14.5 | 2.5 |
| Tetraploid | Kallmyra 3 | 1al | 2 | E7 | 1 | 1.034679 | 3 | -0.66411 | 14 | 2 |
| Tetraploid | Kallmyra 3 | 1al | 3 | S7 | 1 | 0.225614 | 4 | -1.2677 | 14.5 | 0.3 |
| Tetraploid | Kallmyra 3 | 2al | 1A | E- | 0 | -0.64547 | 3 | 0.04523 | 12 | NA |
| Tetraploid | Kallmyra 3 | 2al | 2 | E8 | 0 | 0.819636 | 4 | -1.03822 | 9 | NA |
| Tetraploid | Kallmyra 3 | 3al | 1 | E1 | 0 | -0.40853 | 2 | 2.110377 | 18 | NA |
| Tetraploid | Kallmyra 3 | 3al | 2 | E4 | 1 | -0.44203 | 2 | 1.903789 | 14.16667 | NA |
| Tetraploid | Kallmyra 3 | 4al | 1 | E3 | 1 | 1.44954 | 1 | 1.399619 | 17.75 | 4 |
| Tetraploid | Kallmyra 3 | 4al | 2 | S2 | 1 | 0.583425 | 7 | -0.3608 | 16 | 3.5 |
| Tetraploid | Kallmyra 3 | 4al | 3 | E7 | 1 | -0.6468 | 0 | -0.58023 | 10 | 2 |
| Octoploid | Klippan | 1am | 1 | E1 | 1 | -0.95324 | 3 | 2.810459 | 21.75 | 2 |
| Octoploid | Klippan | 1am | 2 | E4 | 1 | 0.544828 | 4 | 0.248285 | 15.5 | 2.5 |
| Octoploid | Klippan | 2am | 1 | E5 | 1 | -1.1146 | 4 | 0.146121 | 20 | 3 |
| Octoploid | Klippan | 2am | 2 | S5 | 1 | -0.61775 | 3 | 0.1339 | 20 | 1.5 |
| Octoploid | Klippan | 3am | 1 | E4 | 1 | -0.84222 | 0 | 0.715815 | 17 | NA |
| Octoploid | Klippan | 3am | 2 | E3 | 0 | -0.18205 | 4 | 3.94747 | 18 | NA |
| Tetraploid | Kohagen | 1an | 1 | E1 | 0 | 0.239564 | 3 | 2.124369 | 9 | NA |
| Tetraploid | Kohagen | 1an | 2 | S5 | 1 | 1.09543 | 0 | -0.46007 | 14 | NA |
| Tetraploid | Kohagen | 2an | 1 | V | 0 | -0.07 | 2 | -0.61897 | 13.5 | NA |
| Tetraploid | Kohagen | 2an | 2 | E8 | 0 | -1.42319 | 4 | -0.96042 | 13.5 | NA |
| Tetraploid | Kohagen | 2an | 3 | E12 | 0 | -0.73687 | 2 | -0.89275 | 15.5 | NA |
| Tetraploid | Kohagen | 3an | 2 | E10 | 1 | -0.43719 | 5 | -0.24276 | 15.5 | NA |
| Tetraploid | Kohagen | 3an | 3 | E10 | 0 | 0.713406 | 6 | -1.21495 | 13.5 | NA |
| Tetraploid | Kohagen | 4an | 1 | E5 | 0 | 1.236051 | 1 | 1.852783 | 16.33333 | NA |
| Tetraploid | Kohagen | 4an | 2 | E8 | 1 | -1.81197 | 3 | 4.062713 | 15 | NA |
| Tetraploid | Kristinelund 1 | 1ao | 1 | V | 1 | 0.65265 | 2 | 1.522644 | 16 | NA |
| Tetraploid | Kristinelund 1 | 1ao | 2 | E10 | 0 | -0.23255 | 7 | -0.26739 | 14 | NA |
| Tetraploid | Kristinelund 1 | 2ao | 1B | S8 | 1 | -0.97886 | 4 | -0.45542 | 14.5 | NA |
| Tetraploid | Kristinelund 1 | 2ao | 2 | E8 | 0 | 0.487786 | 2 | -2.29375 | 15.5 | NA |
| Tetraploid | Kristinelund 1 | 2ao | 3 | E11 | 0 | 0.565369 | 5 | -0.9979 | 5.5 | NA |
| Tetraploid | Kristinelund 1 | 104ao | 2B | E7 | 1 | 0.205275 | 1 | -0.93843 | 11 | NA |
| Tetraploid | Kristinelund 1 | 104ao | 3 | E- | 1 | 0.926101 | 2 | -2.14607 | 10.5 | NA |
| Tetraploid | Kristinelund 6 | 1ap | 1 | E1 | 0 | -0.35298 | 2 | 1.328062 | 14.5 | NA |
| Tetraploid | Kristinelund 6 | 1ap | 2 | E8 | 1 | 0.601759 | 2 | -0.54844 | 15.5 | NA |
| Tetraploid | Kristinelund 6 | 1ap | 3 | E13 | 1 | -1.60149 | 5 | -1.32204 | 14 | NA |
| Tetraploid | Kristinelund 6 | 2ap | 1 | E7 | 1 | -0.90552 | 3 | 0.734073 | 12 | NA |
| Tetraploid | Kristinelund 6 | 2ap | 2 | E7 | 1 | -0.92221 | 4 | -0.24215 | 12.5 | NA |
| Tetraploid | Kristinelund 6 | 2ap | 3 | S7 | 1 | -0.76632 | 3 | -0.8273 | 14 | NA |
| Octoploid | Kryckeläng | 1aq | 1 | E7 | 1 | 0.292409 | 4 | -0.78031 | 23.5 | NA |
| Octoploid | Kryckeläng | 1aq | 3 | E8 | 0 | -1.28201 | 5 | -0.46382 | 24 | NA |
| Octoploid | Kryckeläng | 2aq | 1 | E7 | 0 | 0.067479 | 3 | -0.21694 | 25 | NA |
| Octoploid | Kryckeläng | 2aq | 2 | E13 | 1 | -2.25399 | 5 | -0.20258 | 24.5 | NA |
| Octoploid | Kryckeläng | 2aq | 3 | E10 | 0 | 0.362774 | 8 | -0.45892 | 20 | NA |
| Octoploid | Kryckeläng | 3aq | 1 | E1 | 0 | -0.74251 | 0 | -1.1132 | 20.5 | NA |
| Octoploid | Kryckeläng | 3aq | 2 | E11 | 0 | -1.38375 | 7 | -0.8089 | 24.5 | NA |
| Octoploid | Kryckeläng | 3aq | 3 | E10 | 0 | 0.572254 | 10 | -0.70329 | 22 | NA |
| Octoploid | Larslund 1 | 1ar | 1 | S2 | 0 | -0.06337 | 3 | -0.88057 | 20 | NA |
| Octoploid | Larslund 1 | 1ar | 2 | E13 | 0 | -0.26525 | 7 | -1.16677 | 24 | NA |
| Octoploid | Larslund 1 | 1ar | 3 | E10 | 0 | -0.49099 | 10 | 0.597877 | 20 | NA |
| Octoploid | Larslund 1 | 2ar | 1 | E8 | 1 | -0.61222 | 3 | -1.54552 | 22.5 | NA |
| Octoploid | Larslund 1 | 2ar | 2 | E11 | 0 | 0.040607 | 7 | -0.65373 | 22 | NA |
| Octoploid | Larslund 1 | 2ar | 3 | E10 | 0 | 1.497604 | 9 | -0.08353 | 24.5 | NA |
| Octoploid | Larslund 1 | 3ar | 2 | E8 | 1 | -0.20067 | 5 | -0.48388 | 22 | NA |
| Octoploid | Larslund 1 | 3ar | 3 | S7 | 0 | -0.98752 | 5 | -0.13283 | 23.5 | NA |
| Octoploid | Larslund 1 | 4ar | 1 | E4 | 1 | -0.8619 | 0 | 1.946538 | 30 | NA |
| Octoploid | Larslund 1 | 4ar | 1/3B | E10 | 0 | -0.60176 | 12 | 0.788513 | 22.5 | NA |
| Tetraploid | Larslund 2 | 1as | 1 | E3 | 0 | 1.874841 | 0 | 0.287631 | 15.5 | NA |
| Tetraploid | Larslund 2 | 1as | 3 | S2 | 1 | 1.431323 | 2 | 0.04907 | 19.5 | NA |
| Tetraploid | Larslund 2 | 2as | 1 | E7 | 1 | -0.9672 | 3 | 0.294151 | 17.6 | 0.3 |
| Tetraploid | Larslund 2 | 2as | 2 | S7 | 1 | -0.53609 | 3 | 0.944452 | 13.875 | 2 |
| Tetraploid | Larslund 2 | 2as | 3 | E8 | 1 | -0.48664 | 3 | 0.946419 | 15 | 3 |
| Tetraploid | Larslund 2 | 3as | 1 | E8 | 0 | -0.16333 | 2 | 0.782335 | 15 | NA |
| Tetraploid | Larslund 2 | 3as | 3 | E10 | 1 | -0.39998 | 7 | -0.11727 | 16 | NA |
| Tetraploid | Larslund 2 | 4as | 1 | E3 | 1 | -0.72477 | 1 | 1.27899 | 15.25 | 0.3 |
| Tetraploid | Larslund 2 | 4as | 2 | S5 | 1 | 0.977324 | 0 | -1.2215 | 15 | 2 |
| Tetraploid | Larslund 2 | 4as | 3 | E13 | 0 | -0.70752 | 5 | -0.80969 | 15.5 | NA |
| Tetraploid | Larslund 3 | 1at | 1 | E7 | 1 | -1.12465 | 4 | 0.167372 | 14 | 2 |
| Tetraploid | Larslund 3 | 1at | 2 | E8 | 1 | -1.03725 | 3 | -0.70096 | 14 | 1 |
| Tetraploid | Larslund 3 | 1at | 3 | E8 | 0 | 1.145959 | 2 | -1.23246 | 15 | NA |
| Tetraploid | Larslund 3 | 2at | 1 | S8 | 1 | -0.11595 | 3 | -0.981 | 12.75 | NA |
| Tetraploid | Larslund 3 | 2at | 2B1 | E10 | 0 | -0.94788 | 9 | -0.73309 | 11.5 | NA |
| Tetraploid | Larslund 3 | 2at | 3 | E10 | 0 | 0.656945 | 3 | -1.53398 | 14.5 | NA |
| Tetraploid | Larslund 3 | 3at | 1A | E- | 1 | -0.68918 | 3 | 2.112355 | 11.75 | 4 |
| Tetraploid | Larslund 3 | 3at | 2 | S8 | 1 | -1.06588 | 3 | 0.781778 | 13.25 | 3.75 |
| Tetraploid | Larslund 3 | 3at | 3 | E8 | 0 | 0.205594 | 3 | 1.841444 | 11.16667 | NA |
| Tetraploid | Larslund 3 | 4at | 1 | E7 | 1 | -1.10216 | 3 | 2.59626 | 13.33333 | NA |
| Tetraploid | Larslund 3 | 4at | 2 | E8 | 0 | 0.52733 | 3 | -0.95695 | 11 | NA |
| Tetraploid | Larslund 3 | 4at | 3 | E11 | 0 | 1.140109 | 4 | -1.37661 | 11.5 | NA |
| Octoploid | Larslund 4 | 1au | 1 | E3 | 1 | -0.04019 | 1 | -0.84409 | 19 | 2 |
| Octoploid | Larslund 4 | 1au | 2 | E6 | 1 | -0.61408 | 3 | 1.008236 | 19.75 | 4 |
| Octoploid | Larslund 4 | 1au | 3 | E8 | 0 | -0.84705 | 5 | 0.080308 | 25 | NA |
| Octoploid | Larslund 4 | 3au | 1 | S2 | 1 | 0.351965 | 3 | -1.11453 | 23 | 1.5 |
| Octoploid | Larslund 4 | 3au | 1B | E6 | 0 | -0.40164 | 4 | -1.57539 | 19.5 | NA |
| Octoploid | Larslund 4 | 3au | 2 | V | 1 | -0.96148 | 3 | 0.450043 | 20 | 3 |
| Octoploid | Larslund 4 | 3au | 3 | E7 | 0 | 0.355419 | 3 | -0.97304 | 22.5 | NA |
| Octoploid | Larslund 4 | 4au | 1 | S3 | 1 | -1.34618 | 4 | -1.01328 | 18.5 | 1 |
| Octoploid | Larslund 4 | 4au | 2 | V | 1 | -0.36212 | 3 | 1.000519 | 23.5 | 3.5 |
| Octoploid | Larslund 4 | 4au | 3 | S8 | 1 | -1.12028 | 5 | -0.87455 | 20.5 | 3 |
| Octoploid | Långbro | 1av | 1 | E8 | 0 | -1.30637 | 4 | -0.19646 | 23 | NA |
| Octoploid | Långbro | 1av | 2 | E8 | 1 | -0.238 | 5 | -0.17866 | 25 | NA |
| Octoploid | Långbro | 1av | 3 | E11 | 0 | -1.75858 | 9 | -1.0352 | 23.5 | NA |
| Octoploid | Långbro | 2av | 1 | S5 | 1 | 1.059007 | 0 | -0.0061 | 21 | 2 |
| Octoploid | Långbro | 2av | 2 | S5 | 1 | -1.68928 | 4 | 0.109943 | 21 | 3 |
| Octoploid | Långbro | 2av | 3 | S7 | 1 | -0.46986 | 5 | -1.50125 | 22 | 0.2 |
| Octoploid | Långbro | 3av | 1 | S2 | 0 | 1.783288 | 3 | 0.490077 | 24.5 | NA |
| Octoploid | Långbro | 3av | 2 | E8 | 0 | -0.76812 | 5 | -0.71357 | 22 | NA |
| Octoploid | Långbro | 3av | 3 | E8 | 0 | -0.06802 | 3 | -0.74626 | 23.5 | NA |
| Octoploid | Långbro | 4av | 1 | S2 | 1 | 0.570895 | 7 | -0.9743 | 19 | 0.3 |
| Octoploid | Långbro | 4av | 2 | E7 | 1 | 0.149261 | 3 | 0.223971 | 20.5 | 3.5 |
| Octoploid | Långbro | 4av | 3 | E8 | 1 | -0.18915 | 4 | -0.3051 | 21.5 | 0.3 |
| Octoploid | N. Ämtvik 1 | 1ay | 1 | S8 | 0 | -1.20188 | 3 | 0.027225 | 20.5 | NA |
| Octoploid | N. Ämtvik 1 | 1ay | 2 | S7 | 1 | -0.66856 | 5 | 0.779051 | 22 | NA |
| Octoploid | N. Ämtvik 1 | 2ay | 1 | S5 | 1 | 0.315933 | 0 | -0.49685 | 21 | 2.5 |
| Octoploid | N. Ämtvik 1 | 2ay | 2A | E- | 1 | -0.69542 | 4 | 1.073185 | 21.5 | 0.2 |
| Octoploid | N. Ämtvik 1 | 2ay | 3 | E8 | 1 | -1.12695 | 5 | -0.00197 | 20.5 | 2 |
| Octoploid | N. Ämtvik 1 | 3ay | 1 | S8 | 1 | -0.61212 | 5 | -0.19895 | 21.5 | 0.3 |
| Octoploid | N. Ämtvik 1 | 3ay | 2A | E- | 1 | -0.37558 | 5 | 0.010964 | 17 | 0.3 |
| Octoploid | N. Ämtvik 1 | 3ay | 3 | E12 | 1 | -1.38598 | 9 | 0.75611 | 19 | NA |
| Octoploid | N. Ämtvik 1 | 4ay | 1 | S5 | 1 | -1.67985 | 2 | 0.635682 | 21 | 3 |
| Octoploid | N. Ämtvik 1 | 4ay | 2 | S7 | 1 | -0.79792 | 5 | -0.40496 | 20 | 4 |
| Octoploid | N. Ämtvik 1 | 4ay | 3 | E11 | 1 | -0.53413 | 9 | -1.50826 | 20 | 0.3 |
| Octoploid | N. Ämtvik 2 | 1az | 1 | E7 | 0 | -0.24877 | 3 | 0.494115 | 19 | NA |
| Octoploid | N. Ämtvik 2 | 1az | 2 | E8 | 1 | -0.93477 | 4 | 1.472348 | 21.5 | NA |
| Octoploid | N. Ämtvik 2 | 2az | 1 | E8 | 0 | -0.22988 | 5 | 0.584332 | 21.5 | NA |
| Octoploid | N. Ämtvik 2 | 2az | 2A | E- | 1 | 1.484417 | 5 | -0.22003 | 20 | 2 |
| Octoploid | N. Ämtvik 2 | 2az | 3 | E8 | 1 | -0.10733 | 5 | 0.353045 | 23.5 | 4.5 |
| Octoploid | N. Ämtvik 2 | 3az | 1 | S8 | 0 | -1.47503 | 5 | 1.659146 | 23 | NA |
| Octoploid | N. Ämtvik 2 | 3az | 2A | S7 | 1 | 1.192925 | 5 | 1.03043 | 23.5 | NA |
| Octoploid | N. Ämtvik 2 | 4az | 1 | E1 | 1 | -0.94007 | 3 | 2.507192 | 23 | 2.5 |
| Octoploid | N. Ämtvik 2 | 4az | 2 | E1 | 1 | -0.50112 | 4 | 2.122666 | 23.5 | 1.5 |
| Octoploid | N. Ämtvik 2 | 4az | 3 | E8 | 1 | -0.40292 | 5 | 0.039719 | 19.5 | 0.2 |
| Octoploid | N. Ämtvik 3 | 2ba | 1 | S7 | 0 | -0.05046 | 4 | -1.26175 | 16.5 | NA |
| Octoploid | N. Ämtvik 3 | 2ba | 2 | E8 | 1 | -0.81063 | 10 | -0.72568 | 18.5 | NA |
| Octoploid | N. Ämtvik 3 | 3ba | 1 | E8 | 0 | 0.05756 | 3 | -0.98159 | 19 | NA |
| Octoploid | N. Ämtvik 3 | 3ba | 2 | E8 | 1 | 1.031327 | 5 | -0.37194 | 19 | NA |
| Octoploid | N. Ämtvik 3 | 3ba | 3 | E13 | 0 | -1.2439 | 9 | -0.04748 | 22 | NA |
| Octoploid | N. Ämtvik 3 | 4ba | 1A | E- | 1 | 0.482883 | 4 | -0.41141 | 19 | NA |
| Octoploid | N. Ämtvik 3 | 4ba | 2 | E10 | 0 | 1.021166 | 11 | -3.19123 | 20.5 | NA |
| Octoploid | Nilslund 1 | 1bb | 1 | S5 | 1 | 0.26869 | 4 | 2.760355 | 23 | 3.5 |
| Octoploid | Nilslund 1 | 1bb | 2 | S5 | 1 | -1.62326 | 3 | 0.364529 | 22 | 3 |
| Octoploid | Nilslund 1 | 3bb | 1 | E1 | 1 | -0.90056 | 3 | 2.818144 | 18 | 1 |
| Octoploid | Nilslund 1 | 3bb | 2 | E3 | 1 | -1.50994 | 3 | 1.543135 | 22 | 0.7 |
| Octoploid | Nilslund 1 | 3bb | 3 | S5 | 1 | -1.14726 | 4 | 0.716822 | 23.5 | 1.5 |
| Octoploid | Nilslund 1 | 4bb | 1 | E4 | 0 | 0.613957 | 1 | -0.26995 | 17 | NA |
| Octoploid | Nilslund 1 | 4bb | 2 | S3 | 1 | -1.45895 | 3 | 2.079824 | 17.5 | NA |
| Octoploid | Nilslund 2 | 1bc | 1A | V | 1 | 0.65265 | 3 | 1.074792 | 23 | NA |
| Octoploid | Nilslund 2 | 1bc | 2 | E7 | 0 | -0.13255 | 4 | 0.175507 | 21.5 | NA |
| Octoploid | Nilslund 2 | 1bc | 3 | E- | 0 | -0.73913 | 4 | -0.67429 | 20.5 | NA |
| Octoploid | Nilslund 2 | 2bc | 1 | E2 | 0 | -1.8078 | 3 | 4.138083 | 21 | NA |
| Octoploid | Nilslund 2 | 2bc | 2 | S4 | 1 | -1.20901 | 3 | 0.473994 | 22.5 | 0.3 |
| Octoploid | Nilslund 2 | 2bc | 3 | E6 | 1 | -0.82652 | 4 | 1.338113 | 23 | 2 |
| Octoploid | Nilslund 2 | 3bc | 1 | E5 | 1 | -1.02057 | 4 | 0.772718 | 23.5 | 2 |
| Octoploid | Nilslund 2 | 3bc | 2 | E7 | 1 | 0.149441 | 8 | 0.795894 | 20 | 2 |
| Octoploid | Nilslund 2 | 3bc | 3 | E7 | 1 | -1.58217 | 4 | 2.963719 | 22 | 4 |
| Octoploid | Nilslund 2 | 4bc | 2 | S7 | 1 | -0.92432 | 5 | 0.214125 | 22 | NA |
| Octoploid | Nilslund 2 | 4bc | 3 | E10 | 0 | 0.562844 | 10 | -1.68152 | 20.5 | NA |
| Octoploid | Norska 1 | 1bd | 1 | E4 | 1 | 0.633635 | 0 | 0.34845 | 20 | 1.5 |
| Octoploid | Norska 1 | 1bd | 2 | E1 | 1 | 0.035877 | 4 | 0.366194 | 21 | 3 |
| Octoploid | Norska 1 | 1bd | 3 | S3 | 0 | 0.611818 | 7 | -1.48028 | 9.5 | NA |
| Octoploid | Norska 1 | 2bd | 1 | E2 | 1 | -0.11253 | 3 | 3.135859 | 20 | 3 |
| Octoploid | Norska 1 | 2bd | 2 | S2 | 1 | -1.03243 | 3 | 1.439062 | 24 | 4 |
| Octoploid | Norska 1 | 2bd | 3 | S4 | 1 | -3.4065 | 5 | 3.409575 | 22 | 5 |
| Octoploid | Norska 1 | 3bd | 1 | E3 | 1 | -1.1041 | 0 | 2.094663 | 21 | 1 |
| Octoploid | Norska 1 | 3bd | 2 | S8 | 1 | -1.09308 | 4 | 0.108534 | 21.5 | 1.5 |
| Octoploid | Norska 1 | 4bd | 1 | E3 | 1 | 1.509938 | 3 | 0.470421 | 23 | 2.5 |
| Octoploid | Norska 1 | 4bd | 2 | S3 | 1 | -0.73222 | 4 | -0.31287 | 17 | 2 |
| Octoploid | Norska 1 | 4bd | 3 | V | 1 | -0.92152 | 4 | -0.53368 | 23 | 3 |
| Tetraploid | Norska 2 | 1be | 1 | S5 | 1 | 0.882506 | 0 | -1.08607 | 12.5 | NA |
| Tetraploid | Norska 2 | 1be | 2 | E3 | 0 | -0.0604 | 4 | -0.97906 | 14 | NA |
| Tetraploid | Norska 2 | 1be | 3 | E4 | 0 | 1.992221 | 1 | -1.13355 | 12 | NA |
| Tetraploid | Norska 2 | 3be | 2 | E10 | 0 | -0.58303 | 11 | -1.17097 | 12 | NA |
| Tetraploid | Norska 2 | 3be | 3 | E8 | 1 | -0.18915 | 3 | -1.95213 | 7.5 | NA |
| Tetraploid | Ryssinge 1 | 1bi | 1 | E4 | 0 | 0.613957 | 0 | 0.810842 | 11 | NA |
| Tetraploid | Ryssinge 1 | 1bi | 2 | S4 | 1 | -1.21828 | 0 | -0.6952 | 10.5 | NA |
| Tetraploid | Ryssinge 1 | 1bi | 3 | E7 | 1 | -0.11623 | 8 | 1.537882 | 13.75 | NA |
| Tetraploid | Ryssinge 1 | 2bi | 1 | E5 | 1 | 0.045054 | 4 | 2.235649 | 11.625 | NA |
| Tetraploid | Ryssinge 1 | 2bi | 3 | S2 | 0 | -0.33772 | 5 | 0.639983 | 10.66667 | NA |
| Tetraploid | Ryssinge 1 | 3bi | 2 | S8 | 1 | 0.824484 | 4 | -1.99685 | 13 | NA |
| Tetraploid | Ryssinge 1 | 3bi | 3 | E8 | 0 | 2.135439 | 3 | -1.67602 | 9.75 | NA |
| Tetraploid | Ryssinge 2 | 1bj | 1 | E3 | 1 | -0.60761 | 0 | 1.841492 | 14.5 | NA |
| Tetraploid | Ryssinge 2 | 1bj | 3 | S2 | 0 | 0.210979 | 5 | 0.777117 | 13.75 | NA |
| Tetraploid | Ryssinge 2 | 2bj | 1 | E3 | 1 | -0.96225 | 0 | -0.35883 | 11.5 | NA |
| Tetraploid | Ryssinge 2 | 2bj | 2 | E3 | 1 | -0.24159 | 2 | 1.937292 | 14.125 | NA |
| Tetraploid | Ryssinge 2 | 2bj | 3A | S5 | 1 | 0.794255 | 3 | 0.247847 | 14.25 | NA |
| Tetraploid | Ryssinge 2 | 2bj | 3 | E8 | 1 | 0.424895 | 4 | -0.30099 | 10.25 | NA |
| Tetraploid | Ryssinge 3 | 1bk | 1A | S1 | 0 | -1.08481 | 3 | 0.047605 | 17.5 | NA |
| Tetraploid | Ryssinge 3 | 1bk | 3 | E13 | 0 | -0.90147 | 11 | -0.32485 | 9.75 | NA |
| Tetraploid | Ryssinge 3 | 2bk | 1 | E1 | 1 | -0.72934 | 0 | 0.286802 | 10 | 3 |
| Tetraploid | Ryssinge 3 | 2bk | 3 | E4 | 1 | -1.00125 | 3 | 1.456486 | 15.25 | 2 |
| Tetraploid | Ryssinge 3 | 3bk | 1 | E1 | 1 | 1.39117 | 1 | 0.623365 | 12 | 2.5 |
| Tetraploid | Ryssinge 3 | 3bk | 2 | S2 | 0 | 0.921733 | 7 | -1.18118 | 12 | NA |
| Tetraploid | Ryssinge 3 | 3bk | 3 | E7 | 1 | -0.30016 | 9 | 1.782831 | 12.75 | 1.5 |
| Tetraploid | Ryssinge 3 | 4bk | 1 | E2 | 1 | 0.071458 | 2 | 1.211603 | 15 | NA |
| Tetraploid | Ryssinge 3 | 4bk | 2 | S5 | 0 | 0.221448 | 4 | 0.180573 | 14 | NA |
| Octoploid | Rågången | 3bl | 2 | V | 1 | -0.16233 | 3 | -1.37128 | 18 | 0.3 |
| Octoploid | Rågången | 3bl | 3 | S8 | 1 | -0.48109 | 4 | -1.8426 | 17.5 | 1.5 |
| Octoploid | Rågången | 39bl | 1A | E7 | 1 | 1.124437 | 3 | -0.7657 | 20 | 3 |
| Octoploid | Rågången | 39bl | 2 | E10 | 1 | -0.54017 | 3 | -0.6182 | 22.5 | 1 |
| Octoploid | Rågången 2 | 1bm | 1 | V | 1 | -1.41036 | 4 | 0.797484 | 22 | 4 |
| Octoploid | Rågången 2 | 1bm | 2 | S7 | 1 | -0.40362 | 5 | -1.14421 | 21.5 | 1.5 |
| Octoploid | Rågången 2 | 2bm | 1 | E4 | 1 | -1.11772 | 0 | 0.96495 | 20.5 | 2.5 |
| Octoploid | Rågången 2 | 2bm | 2 | S2 | 1 | -1.78635 | 2 | -0.33201 | 15 | 3 |
| Tetraploid | Skogstorp 1 | 1br | 1 | S2 | 1 | 1.501716 | 2 | -1.0858 | 13.5 | 0.5 |
| Tetraploid | Skogstorp 1 | 1br | 2 | S2 | 1 | 0.658604 | 7 | -1.32236 | 12.5 | 1.5 |
| Tetraploid | Skogstorp 1 | 2br | 1 | E1 | 1 | 1.127753 | 2 | 0.87903 | 15 | 3.5 |
| Tetraploid | Skogstorp 1 | 2br | 3 | S5 | 1 | -0.706 | 3 | 1.505318 | 13.16667 | 0.3 |
| Tetraploid | Skogstorp 1 | 3br | 1 | S2 | 0 | 0.175195 | 3 | -2.24329 | 12 | NA |
| Tetraploid | Skogstorp 1 | 3br | 2 | V | 0 | -0.88156 | 3 | -1.95956 | 13 | NA |
| Tetraploid | Stene | 1bu | 1 | E4 | 1 | 0.338464 | 0 | 4.386028 | 12.875 | NA |
| Tetraploid | Stene | 1bu | 2 | V | 1 | -1.43583 | 4 | 2.731823 | 12.5 | NA |
| Tetraploid | Stene | 1bu | 3 | E5 | 1 | -0.48776 | 2 | 3.025789 | 14.4 | NA |
| Tetraploid | Stene | 244bu | 1 | S5 | 1 | 1.235508 | 0 | -0.97145 | 13.5 | NA |
| Tetraploid | Stene | 244bu | 2B | E- | 0 | 2.306953 | 1 | -1.55915 | 11.5 | NA |
| Octoploid | Svarv | 1by | 1 | E1 | 0 | -0.5937 | 4 | 1.346076 | 16 | NA |
| Octoploid | Svarv | 1by | 2 | S5 | 1 | -0.44125 | 0 | -0.46844 | 21 | 2 |
| Octoploid | Svarv | 1by | 3 | E7 | 1 | -0.4516 | 3 | -0.1671 | 15.5 | 2.5 |
| Octoploid | Svarv | 2by | 1 | E2 | 0 | 1.254209 | 0 | 1.402915 | 16.5 | NA |
| Octoploid | Svarv | 2by | 2 | S2 | 1 | -1.63362 | 3 | 0.799182 | 22 | NA |
| Octoploid | Svarv | 2by | 3 | S3 | 0 | 0.489219 | 7 | -0.06735 | 21 | NA |
| Octoploid | Svarv | 3by | 1 | E4 | 0 | -0.21176 | 4 | 0.878346 | 15.5 | NA |
| Octoploid | Svarv | 3by | 2 | V | 0 | 1.515889 | 4 | -0.16689 | 18.5 | NA |
| Octoploid | Svarv | 3by | 3 | E6 | 1 | -0.45475 | 4 | 0.295392 | 17 | NA |
| Octoploid | Svarv | 4by | 1 | E5 | 0 | -0.20568 | 4 | 0.401561 | 19.5 | NA |
| Octoploid | Svarv | 4by | 2 | E3 | 1 | 0.24159 | 4 | 2.127942 | 22 | 3 |
| Octoploid | Svarv | 4by | 3 | S4 | 1 | -1.06984 | 4 | 0.21629 | 22 | 1 |
| Tetraploid | Vildhägn 1 | 1cb | 1 | E8 | 0 | -0.14017 | 2 | 0.371652 | 12 | NA |
| Tetraploid | Vildhägn 1 | 1cb | 3 | E10 | 1 | -0.21394 | 7 | -0.89252 | 13.5 | NA |
| Tetraploid | Vildhägn 1 | 75cb | 1 | E7 | 0 | 1.08737 | 1 | -0.99352 | 12.5 | NA |
| Tetraploid | Vildhägn 1 | 75cb | 2 | E7 | 1 | -1.18414 | 3 | -0.43637 | 8 | NA |
| Tetraploid | V-m 1 | 1cd | 1 | E1 | 0 | 1.246291 | 0 | -0.17106 | 12 | NA |
| Tetraploid | V-m 1 | 1cd | 2 | S2 | 1 | -0.18011 | 0 | -1.2432 | 12 | 2 |
| Tetraploid | V-m 1 | 1cd | 3 | E7 | 1 | 0.537734 | 9 | -0.90459 | 12.5 | 2 |
| Tetraploid | V-m 1 | 2cd | 1 | E2 | 1 | 0.767967 | 0 | -0.38873 | 11 | 1 |
| Tetraploid | V-m 1 | 2cd | 2 | E1 | 1 | 0.887661 | 3 | 1.426048 | 14.75 | 3 |
| Tetraploid | V-m 1 | 2cd | 3 | V | 1 | 0.675153 | 6 | -0.001 | 16 | 2 |
| Tetraploid | V-m 1 | 3cd | 1 | E4 | 0 | 0.259751 | 0 | 1.102332 | 7.5 | NA |
| Tetraploid | V-m 1 | 3cd | 2 | S5 | 1 | -0.353 | 4 | 1.363473 | 13.75 | NA |
| Tetraploid | V-m 1 | 3cd | 3 | S3 | 0 | 0.50808 | 9 | -0.12912 | 10.5 | NA |
| Tetraploid | V-m 1 | 4cd | 1 | E2 | 1 | 0.202875 | 0 | 0.487468 | 12.5 | 3 |
| Tetraploid | V-m 1 | 4cd | 1B | V | 1 | -1.4613 | 4 | -0.32617 | 13.25 | 3 |
| Tetraploid | V-m 1 | 4cd | 2 | V | 1 | 0.157333 | 3 | -1.00431 | 14.5 | 2 |
| Octoploid | V-m 2 | 1ce | 1 | S4 | 1 | 0.099156 | 3 | 1.281486 | 18 | NA |
| Octoploid | V-m 2 | 1ce | 3 | E6 | 1 | -0.66719 | 3 | 0.844656 | 17 | NA |
| Octoploid | V-m 2 | 2ce | 1 | E3 | 0 | 0.120795 | 3 | -1.25555 | 14 | NA |
| Octoploid | V-m 2 | 2ce | 2 | V | 0 | -1.48677 | 2 | -3.26431 | 15.5 | NA |
| Octoploid | Ö. Ämtvik 2 | 1cf | 1 | E1 | 0 | -0.46408 | 2 | -1.00175 | 18.5 | NA |
| Octoploid | Ö. Ämtvik 2 | 1cf | 2 | S5 | 1 | 0.174206 | 0 | -0.29403 | 20 | NA |
| Octoploid | Ö. Ämtvik 2 | 1cf | 3 | E13 | 0 | -1.19685 | 8 | -0.77508 | 22.5 | NA |
| Octoploid | Ö. Ämtvik 2 | 2cf | 1A | S8 | 1 | 1.545142 | 4 | -0.60966 | 16 | NA |
| Octoploid | Ö. Ämtvik 2 | 2cf | 2 | E11 | 0 | -0.3842 | 7 | -1.17361 | 16 | NA |
| Octoploid | Ö. Ämtvik 2 | 2cf | 3 | E10 | 0 | 0.62776 | 11 | 0.045445 | 16.5 | NA |

**Table S1-2.** The ploidy type, plant population, plant genet and ramet of the host plant. Larval treatment represents whether the plant was oviposited upon or rejected for oviposition (Yes/No), by the female (rejected plants had a first instar larvae added to it) and final larval size (cm) the size of the larva before disappearing.

| Plant population | Plant genet | Ramet | Ploidy type | Larval treatment | Final larval size |
| --- | --- | --- | --- | --- | --- |
| Almvik | 1a | 1A | Octoploid | No | 0.3 |
| Almvik | 1a | 2 | Octoploid | No | 3.5 |
| Almvik | 2a | 2 | Octoploid | No | 5 |
| Almvik | 2a | 3 | Octoploid | No | 0.3 |
| Almvik | 3a | 1 | Octoploid | No | 0.2 |
| Almvik | 3a | 2A | Octoploid | No | 3 |
| Aspa2 | 2b | 1 | Octoploid | No | 2.25 |
| Aspa2 | 2b | 3 | Octoploid | No | 0.3 |
| Aspa2 | 3b | 1 | Octoploid | No | 1 |
| Aspa2 | 3b | 2 | Octoploid | No | 3.5 |
| Aspa2 | 4b | 1 | Octoploid | No | 3 |
| Aspa2 | 4b | 3 | Octoploid | No | 0.5 |
| AspaIP2 | 1c | 1 | Octoploid | No | 2 |
| AspaIP2 | 1c | 2 | Octoploid | No | 0.3 |
| AspaIP2 | 2c | 2 | Octoploid | Yes | 0.3 |
| AspaIP2 | 3c | 1 | Octoploid | Yes | 2.5 |
| Bysjön | 1g | 3 | Octoploid | No | 4 |
| Bysjön | 2g | 1 | Octoploid | No | 0.5 |
| Bysjön | 2g | 2 | Octoploid | No | 0.3 |
| Bysjön | 3g | 1 | Octoploid | No | 3 |
| Bysjön | 4g | 2A | Octoploid | No | 0.3 |
| Bysjön | 4g | 2B | Octoploid | No | 4 |
| Bysjön | 4g | 3 | Octoploid | No | 0.4 |
| Bölsäter1 | 2h | 1 | Octoploid | No | 0.3 |
| Bölsäter1 | 2h | 1B | Octoploid | No | 4 |
| Bölsäter1 | 3h | 2 | Octoploid | No | 4 |
| Bölsäter1 | 3h | 3 | Octoploid | No | 1 |
| Bölsäter1 | 4h | 1 | Octoploid | No | 2.5 |
| Bölsäter1 | 4h | 2 | Octoploid | No | 3.5 |
| Dammen | 1s | 2 | Octoploid | No | 0.4 |
| Dammen | 1s | 3 | Octoploid | No | 4 |
| Dammen | 2s | 2 | Octoploid | No | 3 |
| Dammen | 3s | 1 | Octoploid | No | 0.3 |
| Dammen | 3s | 2A | Octoploid | No | 3.5 |
| Dammen | 4s | 2 | Octoploid | No | 3 |
| Dammen | 4s | 3 | Octoploid | No | 3 |
| Davik | 1t | 1 | Octoploid | No | 3 |
| Davik | 3t | 1 | Octoploid | Yes | 4 |
| Davik | 3t | 2A | Octoploid | No | 4 |
| Davik | 4t | 1 | Octoploid | No | 3 |
| Davik | 4t | 2 | Octoploid | No | 0.3 |
| Djupbrodal | 1u | 1 | Octoploid | No | 2.75 |
| Djupbrodal | 2u | 3 | Octoploid | No | 0.2 |
| Djupbrodal | 3u | 2 | Octoploid | No | 1 |
| Djupbrodal | 3u | 3 | Octoploid | No | 2 |
| Edeby2 | 1x | 1 | Octoploid | No | 2 |
| Edeby2 | 1x | 2 | Octoploid | No | 3 |
| Edeby2 | 1x | 3A | Octoploid | Yes | 2 |
| Edeby2 | 2x | 2 | Octoploid | No | 0.2 |
| Edeby2 | 3x | 1 | Octoploid | No | 1 |
| Edeby2 | 3x | 2 | Octoploid | No | 3 |
| Edeby2 | 3x | 3 | Octoploid | No | 2 |
| Edeby2 | 4x | 2 | Octoploid | No | 0.4 |
| Edeby2 | 4x | 3 | Octoploid | No | 2 |
| Gustav | 2ad | 3B | Octoploid | No | 2 |
| Gustav | 4ad | 1A | Octoploid | No | 2 |
| Gustav | 4ad | 2 | Octoploid | No | 0.2 |
| Horssjön | 1ae | 1 | Octoploid | No | 0.3 |
| Horssjön | 1ae | 2 | Octoploid | Yes | 0.3 |
| Horssjön | 3ae | 2 | Octoploid | No | 2.5 |
| Horssjön | 4ae | 1 | Octoploid | No | 0.1 |
| Horssjön | 4ae | 2 | Octoploid | No | 2 |
| Horssjön | 4ae | 3 | Octoploid | Yes | 2.5 |
| Inskogsbergen | 1ah | 1 | Octoploid | No | 0.3 |
| Inskogsbergen | 1ah | 2 | Octoploid | No | 3 |
| Inskogsbergen | 1ah | 3 | Octoploid | No | 4 |
| Inskogsbergen | 3ah | 3 | Octoploid | No | 3 |
| Inskogsbergen | 4ah | 1 | Octoploid | No | 4 |
| Inskogsbergen | 4ah | 3 | Octoploid | No | 4 |
| Klippan | 1am | 1 | Octoploid | No | 2 |
| Klippan | 1am | 2 | Octoploid | No | 2.5 |
| Klippan | 1am | 3 | Octoploid | Yes | 0.3 |
| Klippan | 2am | 1 | Octoploid | No | 3 |
| Klippan | 2am | 2 | Octoploid | No | 1.5 |
| Klippan | 2am | 3 | Octoploid | Yes | 2.5 |
| Klippan | 3am | 1 | Octoploid | No | 3 |
| Klippan | 3am | 3 | Octoploid | Yes | 2 |
| Kryckeläng | 1aq | 1 | Octoploid | No | 3 |
| Kryckeläng | 2aq | 2 | Octoploid | No | 4 |
| Larslund1 | 2ar | 1 | Octoploid | No | 2 |
| Larslund1 | 3ar | 2 | Octoploid | No | 2 |
| Larslund1 | 4ar | 1 | Octoploid | No | 3 |
| Larslund4 | 1au | 1 | Octoploid | No | 2 |
| Larslund4 | 1au | 2 | Octoploid | No | 4 |
| Larslund4 | 3au | 1 | Octoploid | No | 1.5 |
| Larslund4 | 3au | 2 | Octoploid | No | 3 |
| Larslund4 | 4au | 1 | Octoploid | No | 1 |
| Larslund4 | 4au | 2 | Octoploid | No | 3.5 |
| Larslund4 | 4au | 3 | Octoploid | No | 3 |
| Långbro | 1av | 2 | Octoploid | No | 1 |
| Långbro | 2av | 1 | Octoploid | No | 2 |
| Långbro | 2av | 2 | Octoploid | No | 3 |
| Långbro | 2av | 3 | Octoploid | No | 0.2 |
| Långbro | 4av | 1 | Octoploid | No | 0.3 |
| Långbro | 4av | 2 | Octoploid | No | 3.5 |
| Långbro | 4av | 3 | Octoploid | No | 0.3 |
| N. Ämtvik1 | 1ay | 2 | Octoploid | No | 1 |
| N. Ämtvik1 | 2ay | 1 | Octoploid | No | 2.5 |
| N. Ämtvik1 | 2ay | 2A | Octoploid | No | 0.2 |
| N. Ämtvik1 | 2ay | 3 | Octoploid | No | 2 |
| N. Ämtvik1 | 3ay | 1 | Octoploid | No | 0.3 |
| N. Ämtvik1 | 3ay | 2A | Octoploid | No | 0.3 |
| N. Ämtvik1 | 3ay | 3 | Octoploid | No | 0.3 |
| N. Ämtvik1 | 4ay | 1 | Octoploid | No | 3 |
| N. Ämtvik1 | 4ay | 2 | Octoploid | No | 4 |
| N. Ämtvik1 | 4ay | 3 | Octoploid | No | 0.3 |
| N. Ämtvik2 | 1az | 2 | Octoploid | No | 2.5 |
| N. Ämtvik2 | 2az | 2A | Octoploid | No | 2 |
| N. Ämtvik2 | 2az | 3 | Octoploid | No | 4.5 |
| N. Ämtvik2 | 3az | 2A | Octoploid | No | 3 |
| N. Ämtvik2 | 3az | 2B | Octoploid | Yes | 3.5 |
| N. Ämtvik2 | 4az | 1 | Octoploid | No | 2.5 |
| N. Ämtvik2 | 4az | 2 | Octoploid | No | 1.5 |
| N. Ämtvik2 | 4az | 3 | Octoploid | No | 0.2 |
| N. Ämtvik3 | 2ba | 2 | Octoploid | No | 1.5 |
| N. Ämtvik3 | 3ba | 2 | Octoploid | No | 3.5 |
| N. Ämtvik3 | 4ba | 1A | Octoploid | No | 3 |
| Nilslund1 | 1bb | 1 | Octoploid | No | 3.5 |
| Nilslund1 | 1bb | 2 | Octoploid | No | 3 |
| Nilslund1 | 3bb | 1 | Octoploid | No | 1 |
| Nilslund1 | 3bb | 2 | Octoploid | No | 0.7 |
| Nilslund1 | 3bb | 3 | Octoploid | No | 1.5 |
| Nilslund1 | 4bb | 2 | Octoploid | No | 4 |
| Nilslund2 | 1bc | 1A | Octoploid | No | 2.8 |
| Nilslund2 | 2bc | 2 | Octoploid | No | 0.3 |
| Nilslund2 | 2bc | 3 | Octoploid | No | 2 |
| Nilslund2 | 3bc | 1 | Octoploid | No | 2 |
| Nilslund2 | 3bc | 2 | Octoploid | No | 2 |
| Nilslund2 | 3bc | 3 | Octoploid | No | 4 |
| Nilslund2 | 4bc | 1 | Octoploid | Yes | 0.3 |
| Nilslund2 | 4bc | 2 | Octoploid | No | 3 |
| Norska1 | 1bd | 1 | Octoploid | No | 1.5 |
| Norska1 | 1bd | 2 | Octoploid | No | 3 |
| Norska1 | 2bd | 1 | Octoploid | No | 3 |
| Norska1 | 2bd | 2 | Octoploid | No | 4 |
| Norska1 | 2bd | 3 | Octoploid | No | 5 |
| Norska1 | 3bd | 1 | Octoploid | No | 1 |
| Norska1 | 3bd | 2 | Octoploid | No | 1.5 |
| Norska1 | 4bd | 1 | Octoploid | No | 2.5 |
| Norska1 | 4bd | 2 | Octoploid | No | 2 |
| Norska1 | 4bd | 3 | Octoploid | No | 3 |
| Rågången | 3bl | 2 | Octoploid | No | 0.3 |
| Rågången | 3bl | 3 | Octoploid | No | 1.5 |
| Rågången | 39bl | 1A | Octoploid | No | 3 |
| Rågången | 39bl | 1 | Octoploid | Yes | 0.3 |
| Rågången2 | 39bl | 2 | Octoploid | No | 1 |
| Rågången2 | 1bm | 1 | Octoploid | No | 4 |
| Rågången2 | 1bm | 2 | Octoploid | No | 1.5 |
| Rågången2 | 2bm | 1 | Octoploid | No | 2.5 |
| Skogstorp1 | 2bm | 2 | Octoploid | No | 3 |
| Svarv | 1by | 2 | Octoploid | No | 2 |
| Svarv | 1by | 3 | Octoploid | No | 2.5 |
| Svarv | 2by | 2 | Octoploid | No | 0.3 |
| Svarv | 3by | 3 | Octoploid | No | 2.5 |
| Svarv | 4by | 2 | Octoploid | No | 3 |
| Svarv | 4by | 3 | Octoploid | No | 1 |
| V-m2 | 1ce | 1 | Octoploid | No | 0.2 |
| V-m2 | 1ce | 3 | Octoploid | No | 5 |

**Table S1-3.** The plant population, ploidy type, final larval size under controlled conditions and host plant use in the wild field populations. Final larval size represents the mean length of larvae grown on genets from a population under controlled conditions. Host plant use represents the mean proportion of oviposited plants within a field population during 2009-2013.

| Population | Ploidy type | Final larval size | Host plant use |
| --- | --- | --- | --- |
| 1 | Tetraploids | 1.63 | 20.67 |
| Bysjön | Octoploids | 1.79 | 54.84 |
| Bölsäter 1 | Octoploids | 2.55 | 23.48 |
| Bölsäter 2 | Tetraploids | 1.83 | 14.36 |
| Dagnäs | Tetraploids | 1.96 | 28.89 |
| Dammen | Octoploids | 2.46 | 10.00 |
| Davik | Octoploids | 2.86 | 40.00 |
| Edeby 1 | Tetraploids | 2.44 | 13.33 |
| Gustav | Octoploids | 1.40 | 8.83 |
| Horssjön | Octoploids | 1.28 | 0.00 |
| Kallmyra 1 | Tetraploids | 1.71 | 27.33 |
| Kallmyra 3 | Tetraploids | 2.08 | 2.22 |
| Kryckeläng | Octoploids | 3.50 | 9.17 |
| Långbro | Octoploids | 1.47 | 5.73 |
| Norska 1 | Octoploids | 2.65 | 11.33 |
| Ryssinge 1 | Tetraploids | 1.82 | 19.33 |
| Ryssing 2 | Tetraploids | 2.21 | 2.00 |
| Ryssinge 3 | Tetraploids | 2.40 | 18.67 |
| Svarv | Octoploids | 1.88 | 8.51 |
| V-m 1 | Tetraploids | 2.11 | 18.33 |
| V-m 2 | Octoploids | 2.60 | 13.50 |
