# Supplementary material for: Butterfly oviposition preference is not related to larval performance on a polyploid herb
Source: Ecol Evol. 2016 Mar 20;6(9):2781–9. doi: 10.1002/ece3.2067 (PMC4863005; doi:10.1002/ece3.2067)
Supplement: Supplementary file 2 — Appendix S2. Models and result tables for ordinary generalized linear models, and generalized linear mixed effects models analyzed in R 3.2.3. Table S2‐1. Egg survival by Anthocharis cardamines in two ploidy types and 51 populations of Cardamine pratensis. Table S2‐2. Larval developments by A. cardamines in two ploidy types and 30 populations of C. pratensis. Table S2‐3. The effect of individual plant traits of two ploidy types of C. pratensis on the development of A. cardamines larvae. Table S2‐4. The effect of oviposition preferences by female A. cardamines on egg survival on two ploidy types of C. pratensis under experimental conditions. Table S2‐5. The effect oviposition preferences by female A. cardamines on larval development on two ploidy types of C. pratensis under experimental conditions. Table S2‐6. Comparison of A. cardamines larval development between larvae feeding on plants chosen for oviposition and larvae feeding on plants rejected for oviposition by butterfly females. Table S2‐7. Correlation between A. cardamines larval performance under experimental conditions and female host plant use in the field for two ploidy types of C. pratensis. [file ECE3-6-2781-s002.doc]

**Supplementary Material Appendix 2**

Butterfly oviposition preference is not related to larval performance on a polyploid herb

**Malin A. E. König^1^, Christer Wiklund^2^, Johan Ehrlén^1^**

^1^Department of Ecology, Environment and Plant Sciences, Stockholm University, SE106 91 Stockholm, Sweden

^2^Department of Zoology, Stockholm University, SE106 91 Stockholm, Sweden 6

**Table of contents**

**Table S2-1-7.** Models and result tables for ordinary generalized linear models, and generalized linear mixed effects models analyzed in R 3.2.3. For each of the examined relationships between fixed factors, and egg survival or final larval length (Table S1 – S6), we ran two types of models. First, we ran ordinary generalized linear models (GLMs) without random effects. Second, we ran generalized linear mixed effect models using the lmer-function in the lme4 package (Bates et al. 2014). The lmer-function allow models including multiple random factors, but does not produce classic P*-*values due to the difficulty associated with calculation of degrees of freedom in mixed effect models. Instead it produces an estimate of the effect of an explanatory variable, its standard error and a t-value or z-value depending on chosen distribution. If the effect estimate ± 1.96 SE does not overlap 0, the variable is regarded to have a significant effect (Baayen et al. 2008). Below, we present the effect of a variable calculated with mixed effect models as: the effect estimate ± 1.96 SE, t-value for general linear models or z-value for generalized linear mixed effect models.

**References**

Baayen RH, Davidson DJ, Bates DM (2008) Mixed-effects modeling with crossed random effects for subjects and items. J Mem Lang 59:390–412 doi: 10.1016/j.jml.2007.12.005

Bates D, Maechler M, Bolker BM Walker S (2014) lme4: Linear mixed-effects models using Eigen and S4. Journal of Statistical Software URL: <http://arxiv.org/abs/1406.5823>

**Table S2-1.** Egg survival by *Anthocharis cardamines* in two ploidy types and 51 populations of *Cardamine pratensis*. (A) GLMs including only the effects ploidy type. (B) Generalized linear mixed effect model with ploidy type used as a fixed factor, and with genet nested within population identity (419 cloned plants nested within 161 genets) and the identity of the butterfly female (n = 21) as random factors. The effect of ploidy type was calculated using octoploids as baseline. The response (egg survival) is binomial and represents if an egg hatched or not (Yes = 1, No = 0).

A) Model: glm(Hatched or not hatched ~ Ploidy type, family= binomial)

|  | Estimate | Std. Error | z-value | P-value |
| --- | --- | --- | --- | --- |
| Ploidy type | 0.20 | 0.21 | 0.96 | 0.34 |

B) Model: glmer(Hatched or not hatched ~ Ploidy type + (1 | Plant population / Plant genet) + (1 | Butterfly female), family= binomial)

| Random factors | Variance | Std. Dev. |
| --- | --- | --- |
| Plant genet : Plant population | <0.001 | <0.001 |
| Plant population | <0.001 | <0.001 |
| Butterfly female | 9.3e-1 | 9.7e-1 |

| Fixed factors | Estimate | Std. Error | z-value |
| --- | --- | --- | --- |
| Ploidy type | 0.25 | 0.22 | 1.13 |

**Table S2-2.**  Larval developments by *A. cardamines* in two ploidy types and 30 populations of *C. pratensis*. (A) GLMs including only the effects ploidy type. (B) Generalized linear mixed effect model with ploidy type is used as a fixed factor, and with genet nested within population identity (173 cloned plants nested within 75 genets) and the identity of the butterfly female (n = 18) as random factors. The effect of ploidy type is calculated using octoploids as baseline. Final larval size represents the last observed length of the larva before the larva abandoned the host plant.

A) Model: glm(Final larval size ~ Ploidy type)

|  | Estimate | Std. Error | z-value | P-value |
| --- | --- | --- | --- | --- |
| Ploidy type | -0.22 | 0.20 | -1.11 | 0.27 |

B) Model: lmer(Final larval size ~ Ploidy type + (1 | Plant population/Plant genet) + (1 | Butterfly female))

| Random factors | Variance | Std. Dev. |
| --- | --- | --- |
| Plant genet : Plant population | <0.001 | <0.001 |
| Plant population | <0.001 | <0.001 |
| Butterfly female | <0.001 | <0.001 |

| Fixed factors | Estimate | Std. Error | t-value |
| --- | --- | --- | --- |
| Ploidy type | -0.22 | 0.20 | -1.11 |

**Table S2-3.** The effect of individual plant traits of two ploidy types of *C. pratensis* on the development of *A. cardamines* larvae. (A) GLMs including the effects ploidy type and three plant traits. (B) Generalized linear mixed effect model with ploidy type and three plant traits used as fixed factors, and with genet nested within population identity (173 cloned plants nested within 75 genets nested within 30 populations) and the identity of the butterfly female (n = 18) as random factors. The effect of ploidy type was calculated using octoploids as baseline. Final larval size represents the last observed length of the larva before the larva abandoned the host plant. Inflorescence size represents the first principle component between log transformed plant mass and square root transformed number of flowers. Phenology represents number of days since first open flower until the plant became oviposited upon.

A) Model: lmer(Final larval size ~ Flower diameter + Inflorescence size + Phenology + Ploidy type + (1 | Plant population / Plant genet) + (1 | Butterfly female))

| Random factors | Variance | Std. Dev. |
| --- | --- | --- |
| Plant genet : Plant population | <0.001 | <0.001 |
| Plant population | <0.001 | <0.001 |
| Butterfly female | 1.5e+00 | 1.2e+00 |

| Fixed factors | Estimate | | Std. Error | | | t-value |
| --- | --- | --- | --- | --- | --- | --- |
| Flower diameter | | 0.01 | | 0.03 | 0.30 | |
| Inflorescence size | | 0.27 | | 0.08 | 3.16 | |
| Phenology | | 0.004 | | 0.05 | 0.07 | |
| Ploidy type | | -0.15 | | 0.39 | -0.38 | |

B) Model: glm(Final larval size ~ Flower diameter + Inflorescence size + Phenology+ Ploidy type)

|  | Estimate | | Std. Error | t-value | P-value |
| --- | --- | --- | --- | --- | --- |
| Flower diameter | | 0.01 | 0.05 | 0.30 | 0.77 |
| Inflorescence size | | 0.27 | 0.08 | 3.16 | 0.0019 |
| Phenology | | 0.004 | 0.05 | 0.07 | 0.94 |
| Ploidy type | | -0.15 | 0.39 | -0.38 | 0.71 |

**Table S2-4.** The effect of oviposition preferences by female *A. cardamines* on egg survival on two ploidy types of *C. pratensis* under experimental conditions. (A) GLMs including the effects of preference and ploidy type. (B) Generalized linear mixed effect model with ploidy type and preference as fixed factors, and with genet nested within population identity (419 cloned plants nested within 161 genets nested within 51 populations) and the identity of the butterfly female (n = 21) as random factors. The effect of ploidy type was calculated using octoploids as baseline. The response (egg survival) is binomial and represents if an egg hatched or not (Yes = 1, No = 0). Oviposition preferences were estimated in a cage experiment as the standardized time from the start of the experiment until a plant received an egg within an experimental trial.

A) Model: glm(Hatched or not hatched ~ Oviposition preferences under controlled conditions * Ploidy type, family=binomial)

|  | Estimate | Std. Error | z-value | P-value |
| --- | --- | --- | --- | --- |
| Preference | -0.06 | 0.16 | -0.35 | 0.73 |
| Ploidy type | 0.22 | 0.21 | 1.03 | 0.31 |
| Preferences : Ploidy type | -0.03 | 0.24 | -0.12 | 0.91 |

B)Model: glmer(Hatched or not hatched ~ Oviposition preferences under controlled conditions * Ploidy type + (1 | Plant population/Plant individual)+ (1 | Butterfly female), family=binomial)

| Random factors | Variance | Std. Dev. |
| --- | --- | --- |
| Plant genet : plant population | <0.001 | <0.001 |
| Plant population | <0.001 | <0.001 |
| Butterfly female | 0.97 | 0.98 |

| Fixed factors | Estimate | Std. Error | z-value |
| --- | --- | --- | --- |
| Preference | -0.04 | 0.18 | -0.22 |
| Ploidy type | 0.27 | 0.23 | 1.17 |
| Preferences : Ploidy type | -0.13 | 0.26 | -0.48 |

**Table S2-5.**  The effect oviposition preferences by female *A. cardamines* on larval development on two ploidy types of *C. pratensis* under experimental conditions. (A) GLMs including the effects of preference and ploidy type. (B) Generalized linear mixed effect model with preference and ploidy type as fixed factors, and with genet nested within population identity (173 cloned plants nested within 75 genets nested in 30 populations) and the identity of the butterfly female (n = 18) as random factors. The effect of ploidy type was calculated using octoploids as baseline. Final larval size represents the last observed length of the larva before the larva abandoned the host plant. Oviposition preferences were estimated in a cage experiment as the standardized time from the start of the experiment until a plant received an egg within an experimental trial.

A) Model: glm(Final larval size ~ Oviposition preferences under controlled conditions * Ploidy type)

|  | Estimate | Std. Error | t-value | P-value |
| --- | --- | --- | --- | --- |
| Preference | -0.03 | 0.15 | -0.19 | 0.85 |
| Ploidy type | -0.23 | 0.21 | -1.09 | 0.28 |
| Preferences : Ploidy type | 0.30 | 0.23 | 1.29 | 0.20 |

B)Model: lmer(Final larval size ~ Oviposition preferences under controlled conditions * Ploidy type + (1 | Plant population / Plant individual) + (1 | Butterfly female))

| Random factors | Variance | Std. Dev |
| --- | --- | --- |
| Plant genet : Plant population | <0.001 | <0.001 |
| Plant population | <0.001 | <0.001 |
| Butterfly female | <0.001 | <0.001 |

| Fixed factors | Estimate | Std. Error | t-value |
| --- | --- | --- | --- |
| Preference | -0.03 | 0.15 | -0.19 |
| Ploidy type | -0.23 | 0.21 | -1.09 |
| Preferences : Ploidy type | 0.30 | 0.23 | 1.29 |

**Table S2-6.**  Comparison of *A. cardamines* larval development between larvae feeding on plants chosen for oviposition and larvae feeding on plants rejected for oviposition by butterfly females. (A) GLMs including the effects of larval treatment (oviposited vs. rejected) and ploidy type. (B) Generalized linear mixed effect model with larval treatment and ploidy type as fixed factors, and with genet nested within population identity (307 cloned plants nested within 161 genets nested within 51 populations) as random factors. The effect of ploidy type was calculated using octoploids as baseline. Final larval size represents the last observed length of the larva before the larva abandoned the host plant. Larval treatment is categorical and corresponds to if the larva was oviposited on the plant or added to a rejected plant later on.

A) Model: glm(Final larval size ~ Larval treatment * Ploidy type)

|  | Estimate | | Std. Error | t-value | P-value |
| --- | --- | --- | --- | --- | --- |
| Larval treatment | | 0.41 | 0.38 | 1.10 | 0.28 |
| Ploidy type | | -0.32 | 0.44 | -0.73 | 0.46 |
| Larval treatment : Ploidy type | | 0.13 | 0.47 | 0.27 | 0.79 |

B)Model: lmer(Final larval size ~ Larval treatment * Ploidy type + (1 | Plant population / Plant individual))

| Random factors | Variance | Std. Dev. |
| --- | --- | --- |
| Plant genet : Plant population | 1.8e-2 | 1.3e-1 |
| Plant population | <0.001 | <0.001 |

| Fixed factors | Estimate | | Std. Error | t-value |
| --- | --- | --- | --- | --- |
| Larval treatment | | 0.41 | 0.38 | 1.10 |
| Ploidy type | | -0.32 | 0.44 | -0.72 |
| Larval treatment : Ploidy type | | 0.12 | 0.47 | 0.25 |

**Table S2-7.**  Correlation between *A. cardamines* larval performance under experimental conditions and female host plant use in the field for two ploidy types of *C. pratensis.* The linear model includes the effects of ploidy type, host plant use and their interaction. Final larval size represents the mean length of larvae grown on genets from a population. Host plant use represents the mean proportion of oviposited plants within a field population during 2009-2013.

Model: lm(Final larval size ~ Host plant use * Ploidy type)

|  | Sum Sq | Df | F-value | P-value |
| --- | --- | --- | --- | --- |
| Host plant use | 0.02 | 1 | 0.08 | 0.79 |
| Ploidy type | 0.21 | 1 | 0.68 | 0.42 |
| Host plant use : Ploidy type | 0.24 | 1 | 0.75 | 0.40 |
